# Type IV secretion system effector sabotages multiple defense systems in a competing bacterium

**DOI:** 10.1093/ismejo/wrae121

**Published:** 2024-07-03

**Authors:** Bingxin Wang, Fugui Xu, Zeyu Zhang, Danyu Shen, Limin Wang, Huijun Wu, Qing Yan, Chuanbin Cui, Pingping Wang, Qi Wei, Xiaolong Shao, Mengcen Wang, Guoliang Qian

**Affiliations:** College of Plant Protection (Key Laboratory of Integrated Management of Crop Diseases and Pests), Nanjing Agricultural University, Nanjing 210095, P.R. China; College of Plant Protection (Key Laboratory of Integrated Management of Crop Diseases and Pests), Nanjing Agricultural University, Nanjing 210095, P.R. China; College of Plant Protection (Key Laboratory of Integrated Management of Crop Diseases and Pests), Nanjing Agricultural University, Nanjing 210095, P.R. China; College of Plant Protection (Key Laboratory of Integrated Management of Crop Diseases and Pests), Nanjing Agricultural University, Nanjing 210095, P.R. China; College of Plant Protection (Key Laboratory of Integrated Management of Crop Diseases and Pests), Nanjing Agricultural University, Nanjing 210095, P.R. China; College of Plant Protection (Key Laboratory of Integrated Management of Crop Diseases and Pests), Nanjing Agricultural University, Nanjing 210095, P.R. China; Department of Plant Sciences and Plant Pathology, Montana State University, Bozeman, MT 59717, United States; Department of Plant Pathology, Shaanxi Provincial Tobacco Corporation of CNTC, Xi'an 710061, China; Department of Plant Pathology, Shaanxi Provincial Tobacco Corporation of CNTC, Xi'an 710061, China; Industrial Crops Institute, Heilongjiang Academy of Agricultural Sciences, Harbin 150086, China; College of Plant Protection (Key Laboratory of Integrated Management of Crop Diseases and Pests), Nanjing Agricultural University, Nanjing 210095, P.R. China; State Key Laboratory of Rice Biology and Breeding, Zhejiang University, Hangzhou 310058, China; Ministry of Agricultural and Rural Affairs Laboratory of Molecular Biology of Crop Pathogens and Insects, Institute of Pesticide and Environmental Toxicology, College of Agriculture and Biotechnology, Zhejiang University, Hangzhou 310058, China; College of Plant Protection (Key Laboratory of Integrated Management of Crop Diseases and Pests), Nanjing Agricultural University, Nanjing 210095, P.R. China

**Keywords:** T4SS, effector, Lysobacter, Pseudomonas, antimicrobial activity, LuxR, PvdS

## Abstract

Effector proteins secreted by bacteria that infect mammalian and plant cells often subdue eukaryotic host cell defenses by simultaneously affecting multiple targets. However, instances when a bacterial effector injected in the competing bacteria sabotage more than a single target have not been reported. Here, we demonstrate that the effector protein, LtaE, translocated by the type IV secretion system from the soil bacterium *Lysobacter enzymogenes* into the competing bacterium, *Pseudomonas protegens*, affects several targets, thus disabling the antibacterial defenses of the competitor. One LtaE target is the transcription factor, LuxR1, that regulates biosynthesis of the antimicrobial compound, orfamide A. Another target is the sigma factor, PvdS, required for biosynthesis of another antimicrobial compound, pyoverdine. Deletion of the genes involved in orfamide A and pyoverdine biosynthesis disabled the antibacterial activity of *P. protegens*, whereas expression of LtaE in *P. protegens* resulted in the near-complete loss of the antibacterial activity against *L. enzymogenes*. Mechanistically, LtaE inhibits the assembly of the RNA polymerase complexes with each of these proteins. The ability of LtaE to bind to LuxR1 and PvdS homologs from several *Pseudomonas* species suggests that it can sabotage defenses of various competitors present in the soil or on plant matter. Our study thus reveals that the multi-target effectors have evolved to subdue cell defenses not only in eukaryotic hosts but also in bacterial competitors.

## Introduction

Microbial pathogens deploy specific effectors that often bind to multiple host targets to simultaneously disrupt various processes in the plant or animal immune systems [1, 2]. For instance, the oomycete RxLR effector Avr3a from *Phytophthora infestans* targets the E3 ligase CMPG1 to suppress INF1-triggered plant cell death [3]. Avr3a also associates with the GTPase dynamin-related protein 2 (DRP2) leading to the suppression of the Pathogen-Associated Molecular Pattern (PAMP) flg22 perception via inhibition of the endocytosis of Pattern Recognition Receptor (PRR) FLS2 [4]. The effector AvrPiz-t from rice blast fungus *Magnaporthe oryzae* manipulates plant immunity and promotes infection by interacting with five host proteins, including two E3 ligases, a bZIP-type transcription factor, a nucleoporin-like protein, and a K^+^-channel protein [5–8]. The type III secretion system (T3SS) effector protein AvrPtoB from the bacterial pathogen *Pseudomonas syringae* binds to several central components of plant immunity mediating their degradation by means of its E3 ligase activity [9]. AvrPtoB-mediated degradation of NPR1 interferes with salicylic acid (SA) signaling [10], whereas degradation of the 8′-hydroxylase CYP707A of the abscisic acid (ABA) signaling pathway promotes ABA accumulation [11]. Furthermore, *Helicobacter* effector protein, CagA, delivered by the type IV secretion system (T4SS) binds to several proteins of the gastric mucosal epithelial cells [12, 13], including Grb2 and PAR1/MARK, to induce inflammation and promote epithelial-mesenchymal transition. Whereas the ability of effectors from microbial pathogens to disable multiple targets in the plant and mammalian cells has been established, it has not been observed in the competition among bacterial cells.

In this study, we focused on bacteria from the *Lysobacter* and *Pseudomonas* genera known to be ubiquitous in soil [14–17]. Consequently, members of these genera are likely to encounter each other and engage in the interspecies interactions. *Lysobacter enzymogenes* OH11 synthesizes and secretes chemicals that inhibit fungal pathogens [18]. It also possesses a T4SS capable of injecting various effector proteins into bacterial competitors. These effectors were shown to disrupt various processes in the competitor cells, inducing cell death and quorum quenching [19, 20]. *Pseudomonas protegens* Pf-5 is another beneficial soil bacterium that produces various antimicrobial compounds targeting its fungal and bacterial competitors. Under laboratory conditions, the antifungal and antibacterial properties of *P. protegens* are manifested in the King’s B (KB) medium [21, 22] but not in Potato Dextrose Agar (PDA), a medium commonly used for fungal growth. However, when *P. protegens* encounters *L. enzymogenes*, its antifungal activity is activated in the PDA medium [23] as a result of the translocation of the *L. enzymogenes* T4SS effector protein, LtaE, into the cytoplasm of *P. protegens*. LtaE binds the *P. protegens* PhlF protein that functions as a transcription repressor of the antifungal 2,4-diacetylphloroglucinol (2,4-DAPG) biosynthetic operon, impairing its repressor function and stimulating 2,4-DAPG production [23, 24].

Here we discovered that, in addition to targeting PhlF, *L. enzymogenes* LtaE interacts with two additional *P. protegens* proteins, LuxR1 and PvdS. LuxR1 is a pathway-specific transcriptional regulator of biosynthesis of the antimicrobial compound orfamide A, whereas PvdS is a sigma factor controlling biosynthesis of pyoverdine. Both orfamide A and pyoverdine are toxic for *L. enzymogenes*. Therefore, the injected LtaE sabotages synthesis of both compounds in *P. protegens*. Because *Lysobacter* LtaE effectors interact with LuxR1 and PvdS homologs from multiple *Pseudomonas* species, it appears that the observed mechanism is common. We conclude that translocating multifunctional effectors that disabling antimicrobial defense systems is not exclusive to eukaryotic hosts but also operates in bacterial competitions.

## Materials and methods

### Bacterial strains, plasmids, and growth conditions

The bacterial strains and plasmids used in this work are listed in Table S1. Unless otherwise stated, all tested bacteria and their derivatives were grown in Luria–Bertani (LB) media for bacterial revival and initial cultivation at 28°C or 37°C (for *Escherichia coli* strains), KB medium was employed for subculturing or bioassays. The following antibiotics were added when necessary: Ampicillin (Amp) at 100 μg/mL, tetracycline (Tet) at 50 μg/mL, kanamycin (Km) at 50 μg/mL, gentamicin (Gm) at 100 μg/mL, and chloramphenicol (Cm) at 100 μg/mL. Bacterial growth was determined by measuring optical density at a wavelength of 600 nm (OD_600_).

### Genetic methods

We utilized a double-crossover homologous recombination strategy to generate in-frame deletion mutants in both *L. enzymogenes* and *P. protegens*, following the previously outlined method [23]. In brief, DNA fragments flanking the target genes were PCR-amplified using specific primers (Table S2) and then integrated into broad host suicide vectors—pEX18Gm for *L. enzymogenes* and pK18mobSacB for *P. protegens* (Table S1). The resulting recombinant vectors were separately introduced into *L. enzymogenes* and *P. protegens* through electroporation. Single-crossover recombinants were initially selected on LB medium supplemented with 1.6% agar (LA), along with Km and Gm for *L. enzymogenes* or Km and Amp for *P. protegens*. Subsequently, transformants underwent further screening via double-crossover selection, cultivating them on LA plates containing 10% (w/v) sucrose. Confirmation of mutants was performed via PCR analysis employing specific primers (Table S2).

The identical double-crossover homologous recombination approach, employing the primers outlined in Table S2, was utilized to facilitate the generation of chromosomal gene insertions. Briefly, PCR was employed to amplify intact genes, which were subsequently inserted into the suicide vectors pEX18Gm for *L. enzymogenes* or pK18mobSacB for *P. protegens*. The selection process followed the above-described protocol.

### Protein expression and purification

Protein expression and purification were conducted following established protocols [25]. In brief, the LtaE protein was expressed as a Glutathione S-transferase (GST) fusion, whereas LuxR1 and PvdS proteins were expressed as N-terminal His-Trigger Factor (TF) fusions. Subsequently, these proteins were purified using affinity chromatography. The coding sequences of LuxR1 and PvdS were integrated into the pCold TF plasmid (No. 3365, Takara, Japan), and the coding region of LtaE was inserted into the pGEX-6P-1 plasmid, utilizing the primers detailed in Table S2. These plasmids were then introduced into *E. coli* BL21(DE3) strains (Table S1). The His-fusion proteins were isolated from 400 mL of *E. coli* BL21(DE3) cultures employing Ni-NTA resin (GE Healthcare, Shanghai, China), whereas the GST-fusion proteins were purified from an equivalent volume of *E. coli* BL21(DE3) cultures using GST resin (GE Healthcare, Shanghai, China). Bacterial cultures were cultivated until they reached an optical density at 600 nm (OD_600_) of 0.6 at 37°C. Subsequently, they were induced with 0.4 mM isopropyl β-D-1-thiogalactopyranoside (IPTG, Sigma, USA) at 16°C for a duration of 16 h. The purity of the proteins was assessed through Sodium Dodecyl Sulfate-Polyacrylamide Gel Electrophoresis (SDS-PAGE), and the protein concentration was determined using a BCA protein assay kit (Sangon Biotech, Shanghai, China).

### Pull-down assays

The pull-down assays followed an established protocol [23]. Equal volumes of His-tagged and FLAG-tagged proteins were combined in 1 mL of 1 × PBS buffer (P1020, Solarbio, China). Next, the mixture was supplemented with 20 μL of anti-FLAG magnetic beads (Bimake, Shanghai, China) and left to incubate overnight at 4°C. The magnetic beads were collected at 4°C and washed five times with 1 × PBS buffer containing 1% Triton X-100 to remove nonspecifically bound proteins. Proteins captured on FLAG-beads were eluted by boiling with 4× SDS loading dye for 8 min, followed by SDS-PAGE and western blotting. Protein detection employed specific FLAG-(M20008S) and His-(ab18184) antibodies (Abmart, Shanghai, China).

### Microscale thermophoresis assay

Protein–protein binding affinities were assessed using the Monolith NT.115, as described previously [23]. The His-TF-LuxR1 protein was fluorescently labeled with RED-Tris-NTA dye (MO-L018). A constant concentration of labeled protein (20 nM) in MST buffer was titrated against GST-LtaE or GST proteins over a range from 0.38 nM to 25 μM. MST premium-coated capillaries (MO-K022) were employed to introduce samples into the MST instrument at 25°C, using medium MST power and 100% LED power. Laser intervals were set at 10-s intervals for activation and deactivation. The interaction between His-TF-PvdS and GST-LtaE followed the same protocol.

For the competitive LuxR binding assay involving LtaE and *E. coli* RNA polymerase core enzyme (NEB), the LuxR1-His protein was labeled with RED-Tris-NTA dye (MO-L018). A fixed concentration of LuxR1 protein (20 nM) in MST buffer was titrated against *E. coli* RNA polymerase core enzyme, ranging from 15.2 pM to 1 μM. In the competitive binding assay, the labeled LuxR1-His protein was incubated with GST-LtaE protein at 4°C for 10 min, following the same procedure for the control GST protein. MST premium-coated capillaries (MO-K022) were used to load the samples into the MST instrument at 25°C, employing medium MST power and 100% LED power. All experiments were conducted in triplicate and analyzed using NanoTemper Analysis v2.3 software (NanoTemper Technologies). The identical protocol was applied for the competitive PvdS binding assay between LtaE and *E. coli* RNA polymerase core enzyme.

### Orfamide A extraction and quantification

Orfamide A was purified using a previously established method [26]. In brief, *Pseudomonas* strains were cultured in 20 mL of KB liquid medium and agitated for 40 h at 28°C in a shaking incubator. Following centrifugation, the supernatant was collected and acidified to pH 2.0 using hydrochloric acid. Subsequently, it was allowed to precipitate at 4°C for 12 h. After centrifugation at 4°C (12 000 rpm for 10 min), the supernatant was discarded, and the precipitate was collected. The precipitate was dissolved in 20 mL of methanol, filtered through a 0.22 μm filter, and subjected to HPLC analysis with a detection wavelength of 202 nm. Quantification of orfamide A was accomplished by measuring the peak areas on the HPLC chromatograms and normalizing them to the optical density at 600 nm (OD_600_) of the culture.

### Pyoverdine detection assays

Pyoverdine detection assays in *Pseudomonas* species were conducted following established protocols [27]. The test strains were cultured overnight in KB medium and then sub-cultured at a 1:100 ratios in iron-deficient SSA medium (K_2_HPO_4_ 6 g/L; KH_2_PO_4_ 3 g/L; (NH_4_)_2_SO_4_ 1 g/L; MgSO_4_ 0.1 g/L; succinic acid 4 g/L; pH 7.0). After 48 h of incubation at 28°C, the supernatant was collected through centrifugation, and fluorescence was measured using either a UV irradiation platform or a multifunctional microplate reader. Fluorescence values were recorded with an excitation wavelength of 405 nm and an emission wavelength of 480 nm.

### Promoter activity assays

The assessment of promoter activity in *P. protegens* followed established protocol [23]. Primers specified in Table S2 were employed for amplifying the promoter region. The resultant PCR fragments underwent purification before being cloned into the promoterless luciferase reporter vector pMS402 (Table S1). These modified vectors were then introduced into both the wild-type Pf-5 and its derivatives. To conduct luciferase assays in a liquid medium, cultures of the wild-type Pf-5 or its derivatives containing each promoter reporter vector were incubated in liquid KB at 28°C overnight. Subsequently, two microliters of the culture were transferred into a black transparent 96-well flat-bottom microplate (No. 3904, CORNING, USA) filled with 200 μL of KB liquid medium. Luminescence and OD_600_ absorbance values were recorded at 15-min intervals using a multifunctional microplate detector (Synergy H1, Biotek, USA).

### Bacterial two-hybrid assay

The BacterioMatch II two-hybrid (B2H) system (Agilent Technologies, USA) was employed to detect protein–protein interactions [19]. In brief, the coding region of LtaE and its homologs was cloned into the pBT plasmid, whereas the coding region of the target proteins was inserted into the pTRG plasmid. These constructs were transformed into the *E. coli* XL1-Blue MRF’Kan strain. Positive controls included plasmids pBT-GacS and pTRG-GacS (Table S1), whereas negative controls comprised transformants with empty pTRG and pBT vectors. Selective medium was applied to each co-transformant and incubated at 28°C for 2–3 days. Successful interaction between the two proteins was determined by the growth of transformed XL1-Blue MRF’Kan strain carrying both vectors on histidine-deficient medium supplemented with 5 mM 3-amino-1,2,4-triazole (3-AT) and 2 μg/mL streptomycin (Str). LB agar, a non-selective medium supplemented with 12.5 μg/mL Tet, 34 μg/mL Cm, and 30 μg/mL Km, was employed to ensure successful transformation of both vectors into the XL1-Blue MRF’Kan strain.

### Protein co-localization assays by fluorescence microscopy

Protein co-localization assays were conducted as previously described [23]. To validate the co-localization of LtaE with LuxR1 and PvdS in *E. coli*, the pBAD/MYC-His A vector was used to express green fluorescent protein (GFP) and LtaE-GFP fusion protein, whereas the pBADGM vector was employed to express red fluorescent protein (mCherry), LuxR1-mCherry fusion proteins, and PvdS-mCherry fusion proteins, respectively. After co-transforming these constructed combinations of green and red fluorescent protein vectors into *E. coli* BL21(DE3), the bacterial strains were cultured until reaching an OD_600_ of 0.4 at 37°C, then induced for 2 h at 28°C with the addition of 0.2% arabinose. Subsequently, the bacterial strains expressing green and red fluorescence were observed using an inverted fluorescence microscope (Axio Observer 3, Zeiss, Germany) with a 63× oil immersion objective. Images were captured and analyzed using ZEN 3.2 software (Zeiss).

## Results

### *Lysobacter enzymogenes* LtaE interacts with transcription regulators of two antibacterial systems in a competing bacterium *P. protegens*

**Figure 1:**
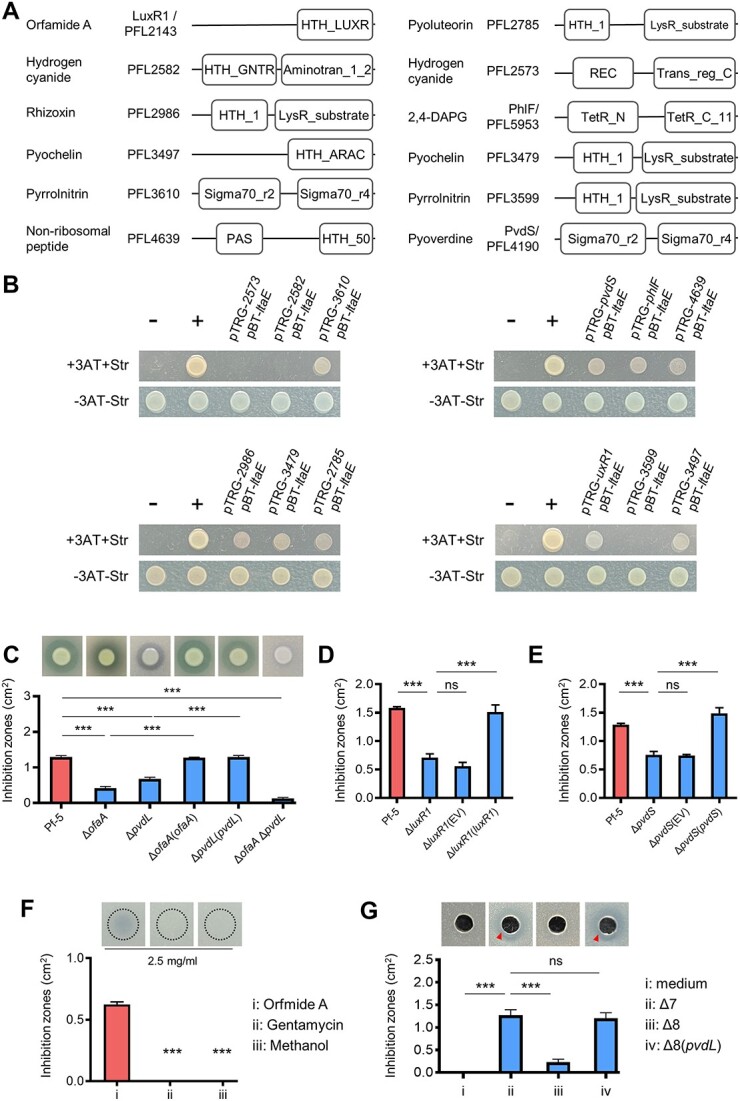
**LtaE interacts with transcription regulators of two antibacterial systems in a competing bacterium.** (**A**) Schematic model of 12 pathway-associated transcriptional regulators within nine gene clusters/operons responsible for biosynthesis of antimicrobial metabolite in *P. protegens* Pf-5. (**B**) Bacterial two-hybrid assays reveal the interaction between LtaE and the pathway-associated regulators shown in (A). Interaction between the two proteins was determined by the growth of transformed *E.coli* strain carrying both vectors on histidine-deficient medium supplemented with 5 mM 3-AT and 2 μg/mL Str. “-” represents the negative control; “+” represents the positive control; “+3AT + Str” indicates the addition of 3-AT and Str; “-3AT-Str” indicates the absence of 3-AT and Str. (**C**) Individual or in-combination mutation of *ofaA* and *pvdL*, the respective biosynthetic genes of orfamide A and pyoverdine, impaired the antibacterial activity of *P. protegens* against *L. enzymogenes* on KB plates. The wild-type strain OH11 of *L. enzymogenes* was embedded in KB plates and *P. protegens* was inoculated on the plate surface. Inhibition zones were observed after 48-h incubations and the zone areas were calculated for quantification. Δ*ofaA*, a mutant strain with an in-frame deletion of *ofaA* that is the first gene of orfamide A biosynthetic operon. Δ*ofaA*(*ofaA*), a strain with chromosomal complementation of *ofaA* in Δ*ofaA.* Δ*pvdL*,a mutant strain with an in-frame deletion of *pvdL* that is a key member of pyoverdine biosynthetic operons. Δ*pvdL*(*pvdL*), a strain with chromosomal complementation of *pvdL* in Δ*pvdL*. Δ*ofaA* Δ*pvdL*, a double mutant of *ofaA* and *pvdL*. (**D–E**) Mutation of *luxR1* (**D**) or *pvdS* (**E**) in *P. protegens* Pf-5 decreased the antibacterial activity against the growth of *L. enzymogenes* OH11. Δ*luxR1*, an in-frame deletion mutant of *luxR1* at the wild-type background of *P. protegens* Pf-5. Δ*luxR1*(*luxR1*), Δ*luxR1* carrying a plasmid-borne *luxR1*. Δ*pvdS*, an in-frame deletion mutant of *pvdS* at the wild-type background of *P. protegens* Pf-5. Δ*pvdS*(*pvdS*), Δ*pvdS* carrying a plasmid-borne *pvdS*. EV, empty vector control. (**F**) Antibacterial test of the commercial orfamide A standard against the growth of *L. enzymogenes* OH11. Gentamycin at the same concentration and methanol were used as controls. The dashed circle indicates the initial location for adding the sample. (**G**) The pyoverdine-containing cell-free supernatant produced by *P. protegens* directly inhibited the growth of *L. enzymogenes* OH11. Δ7, a strain Pf-5-derived mutant with in-frame deletion of seven key genes (*phlD*, *pltB*, *ofaA*, *prnA*, *hcnABC*, *PFL4656*, and *rzxB*) within respective gene clusters/operons responsible for antimicrobial metabolite biosynthesis. Δ8, in-frame deletion of *pvdL* in Δ7, Δ8(*pvdL*), a *pvdL* chromosomally complemented strain in Δ7. The triangle arrow indicates the antagonistic region. In panels (**C–G)**, the results were presented as the mean ± standard deviation (SD) from three independent biological replicates (*n* = 3). Statistical analysis was performed using one-way ANOVA with Tukey’s multiple-comparison test. ^*^^*^^*^P < 0.001; ns, not significant.

In our earlier work exploring the *L. enzymogenes*—*P. protegens* encounters, we observed that the *L. enzymogenes* T4SS effector, LtaE, secreted in the cytoplasm of the strain *P. protegens* Pf-5, binds the transcriptional repressor, PhlF, and derepresses biosynthesis of the antifungal agent, 2,4-DAPG [23]. We posed the question of whether LtaE can also interact with transcriptional regulators of other secondary metabolite biosynthesis pathways in this strain [22]. We identified regulators because they are commonly located within the biosynthesis gene clusters [28–30]. By using a bacterial two-hybrid (B2H) assay, we found that nine potential transcription regulators bound to LtaE (Fig. 1A and B). In the B2H assays, the growth of *E. coli* on this selective medium indicated a direct interaction between LtaE and the examined transcription regulators. Conversely, the failure of the *E. coli* strains to grow on the selective medium suggested an absence of protein–protein interactions.

To understand the significance of LtaE recognizing multiple pathway-associated regulators in *P. protegens*, we investigated if products of these biosynthetic clusters affect *L. enzymogenes*. We selected previously constructed or generated in-frame deletions in key biosynthetic genes within each of the nine gene clusters (Fig. S1). Two mutants, Δ*ofaA* and Δ*pvdL*, showed weaker antibacterial activity against *L. enzymogenes* as judged by the smaller inhibition zones on KB plates, compared to zones formed by the wild-type *P. protegens* (Fig. 1C, Fig. S2). *ofaA* and *pvdL* belong to the orfamide A and pyoverdine biosynthesis clusters, respectively. The antibacterial potency of the Δ*ofaA* or Δ*pvdL* mutants was restored by the chromosomal knock-in of the wild-type *ofaA* or *pvdL* genes, respectively. The double mutant, Δ*ofaA* Δ*pvdL,* almost completely lost its ability to inhibit *L. enzymogenes* growth on the KB plates almost completely (Fig. 1C).

Based on the ability of LtaE to interact with the transcription regulators of the orfamide A and pyoverdine biosynthesis gene clusters, LuxR1 and PvdS, and on the inhibitory activities of the products of these clusters against *L. enzymogenes*, we hypothesized that LtaE may sabotage the antibacterial activity of *P. protegens*. To test this hypothesis, we needed to establish that LuxR1 and PvdS are important for the antibacterial activity. Consistent with our expectation, the in-frame deletions in *luxR1* and *pvdS*, Δ*luxR1* and Δ*pvdS*, respectively, significantly compromised the antibacterial activity of *P. protegens*. The antibacterial activity of each mutant was restored upon the introduction of the wild-type *luxR1* or *pvdS* gene (Fig. 1D and E). The growth in liquid KB medium of the generated *P. protegens* mutants was not impaired (Figs S3 and S4), therefore mutations in these genes are specific to antibacterial activity. We conclude that by interacting with two protein targets in *P. protegens*, LuxR1 and PvdS, *L. enzymogenes* LtaE sabotages the antibacterial activity of its competitor.

We wanted to verify the toxicity of orfamide A and pyoverdine to *L. enzymogenes*. Toxicity of orfamide A on *L. enzymogenes* could be verified directly, by using the commercially available product (Fig. 1F). However, a direct antibacterial test of pyoverdine was not feasible because the structure of the *P. protegens* pyoverdine remains unknown. We therefore undertook an alternative approach, using the pyoverdine-containing culture supernatant. For this experiment, we used the previously constructed *P. protegens* mutant strain, Δ7 (*phlD*, *pltB, ofaA, prnA, hcnABC, PFL4656,* and *rzxB*) [23] that contains in-frame deletions in seven biosynthesis gene clusters, including orfamide A, but not pyoverdine. The cell-free medium of the *P. protegens* Δ7 mutant inhibited *L. enzymogenes* growth. In contrast, the culture medium of the constructed strain Δ8, which has an additional mutation of *pvdL* in strain Δ7, did not inhibit *L. enzymogenes* growth. The antibacterial defect of strain Δ8 was restored by the chromosomal knock-in insertion of the *pvdL* gene, Δ8(*pvdL*) (Fig. 1G). Consistent with the presented results, pyoverdine was detected in the cell-free culture supernatants [31, 32] of strains Δ7 and Δ8(*pvdL*), but not Δ8 (Fig. S5a–b). The growth curves of the mutant and complemented strains were similar to those of the wild type (Fig. S6). Altogether, these data support the notion that orfamide A and pyoverdine possess activities detrimental to *L. enzymogenes*.

### LuxR1 and PvdS are pathway-specific regulators of biosynthesis of orfamide A and pyoverdine, respectively

**Figure 2:**
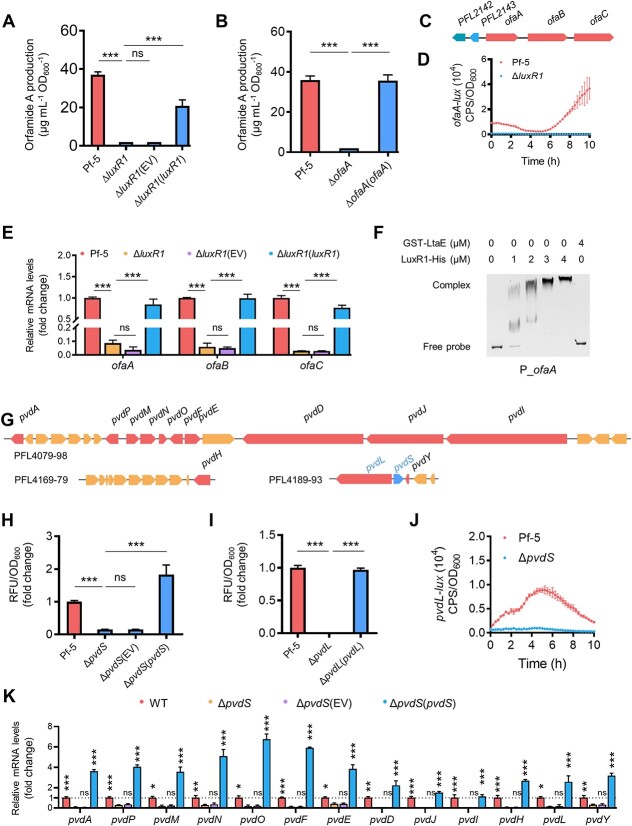
**LuxR1 and PvdS serve as transcriptional regulators associated with the pathways of orfamide A and pyoverdine biosynthesis in *P. protegens*.** (**A–B**) HPLC-based quantification of orfamide A yield produced by Δ*luxR1* (**A**) and Δ*ofaA* (**B**). Δ*luxR1*, an in-frame deletion mutant of *luxR1* at the wild-type background of *P. protegens*. Δ*luxR1*(*luxR1*), Δ*luxR1* carrying a plasmid-borne *luxR1*. Δ*luxR1*(EV), Δ*luxR1* carrying an empty vector. Δ*ofaA*, a mutant strain with an in-frame deletion of *ofaA* that is the first gene of orfamide A biosynthetic operon. Δ*ofaA*(*ofaA*), a strain with chromosomal complementation of *ofaA* in Δ*ofaA*. (**C**) Schematic illustration of the *ofaABC* operon. PFL2143 codes for LuxR1. (**D**) Monitoring of *ofaA*-*lux* activity in Δ*luxR1* in liquid KB. (**E**) Relative mRNA expression (fold change) of the *ofaABC* operon genes analyzed by qRT-PCR. (**F**) EMSA showed LuxR1-His directly bound to the promoter region of the *ofaABC* operon. (**G**) Schematic illustration of pyoverdine biosynthetic operon genes in *P. protegens* Pf-5. (**H–I**) Fluorescence-based quantification of pyoverdine yield (fold change) produced by Δ*pvdS* (**H**) and Δ*pvdL* (**I**)*.* Δ*pvdS*, an in-frame deletion mutant of *pvdS* at the wild-type background of *P. protegens* Pf-5. Δ*pvdS*(*pvdS*), Δ*pvdS* carrying a plasmid-borne *pvdS*. Δ*pvdS*(EV), Δ*pvdS* carrying an empty vector. Δ*pvdL*, a mutant strain with an in-frame deletion of *pvdL* that is a key member of pyoverdine biosynthetic operons. Δ*pvdL*(*pvdL*), a strain with chromosomal complementation of *pvdL* in Δ*pvdL*. (**J**) Monitoring of *pvdL*-*lux* activity in Δ*pvdS* in liquid KB. (**K**) Relative mRNA expression (fold change) of the 13 pyoverdine operon genes analyzed by qRT-PCR. In panels (A), (B), (E), (H), (I), and (K), results were presented as mean ± SD from three independent biological replicates (*n* = 3). One-way ANOVA with Tukey’s multiple-comparison test was conducted. ^*^^*^^*^P < 0.001; ^*^^*^P < 0.01; ^*^P < 0.05; ns, not significant.

Whereas our predictions of the roles of validate LuxR1 and PvdS as pathway-specific regulators of orfamide A and pyoverdine biosynthesis were consistent with the mutant analysis presented above, experimental validation was lacking. We therefore conducted genetic and biochemical assays to establish such relationships. Orfamide A was detected by HPLC in the cell-free media of the wild-type strain, Pf-5, but not the Δ*luxR1* or Δ*ofaA* mutants (Fig. 2A and B and Figs S7 and S8). The orfamide A production in the Δ*luxR1* and Δ*ofaA* mutants could be rescued by introduction of the respected wild-type genes (Figs 2A, B, S7 and S8). These results provide compelling evidence that LuxR1 acts as an activator of orfamide A biosynthesis.

We assessed the role of LuxR1 in transcription of the orfamide A biosynthesis operon, *ofaABC*. To this end, we engineered an *ofaABC* promoter-driven luciferase (*luxCDABE*) reporter, *ofaA-lux* (Fig. 2C). We found that the *ofaA*-*lux* expression in the *luxR1* mutant was reduced (Fig. 2D). Corroborated this finding, the quantitative reverse transcription PCR (qRT-PCR) assays demonstrated a significant reduction in *ofaA*, *ofaB*, and *ofaC* mRNA levels in the Δ*luxR1* mutant. Introduction of the plasmid-borne *luxR1* restored *ofaABC* transcription levels to those of the wild type (Fig. 2E). Furthermore, the electrophoretic mobility shift assay (EMSA) showed that the purified LuxR1-His fusion protein bound to the promoter region upstream of *ofaA* (Fig. 2F). These results establish that LuxR1 acts as a transcription activator of the *ofaABC* operon.

A similar strategy to LuxR1 was employed to assess the role of PvdS. In *Pseudomonas aeruginosa*, PvdS acts as an RNA polymerase sigma factor required for pyoverdine biosynthesis [33, 34]. It was therefore reasonable to assume that PvdS plays the same role in *P. protegens* (Fig. 2G). Pyoverdine production in the Δ*pvdS* mutant was inhibited nearly completely (Fig. 2H), similar the mutation in the biosynthesis gene, *pvdL* (Fig. 2I). Pyoverdine levels in the *pvdS* mutant were restored to the wild-type levels by the plasmid-expressed *pvdS* gene (Fig. 2H). The expression of the *pvdL* promoter-luciferase fusion, *pvdL-lux*, was significantly reduced in the Δ*pvdS* mutant, compared to the wild type (Fig. 2J). This was corroborated by the decrease pyoverdine biosynthesis gene transcripts measured by qRT-PCR. The plasmid-borne *pvdS* gene rescued the transcriptional defects of the Δ*pvdS* mutant (Fig. 2K). These findings support the role of PvdS as a transcription activator, likely a sigma factor, controlling pyoverdine biosynthesis in *P. protegens*.

The orfamide A biosynthesis operon and *luxR1* are present in many *Pseudomonas* species (Fig. S9). The same is true for the pyoverdine biosynthesis gene cluster and *pvdS* (Fig. S10). Therefore, *L. enzymogenes* LtaE may inhibit orfamide A and pyoverdine synthesis in various *Pseudomonas* species.

### LtaE specifically binds LuxR1 and PvdS

**Figure 3:**
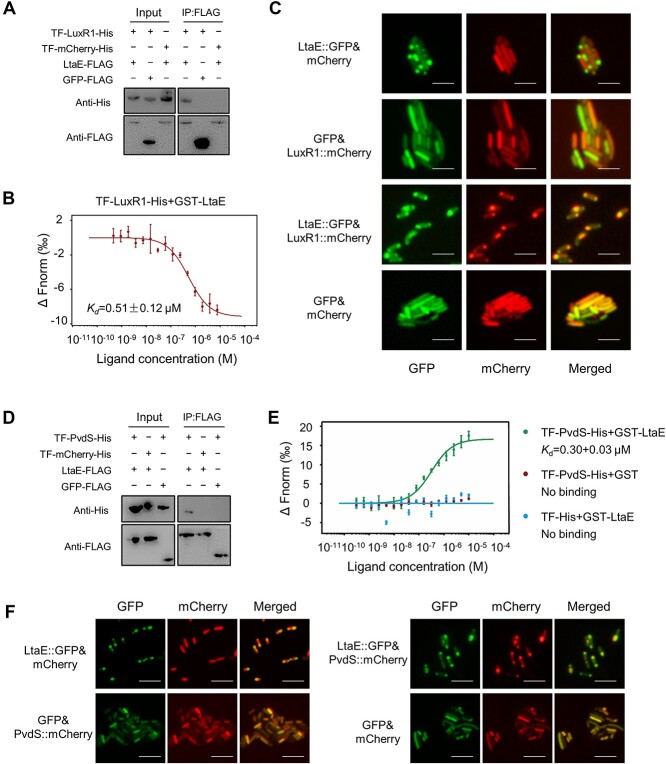
**LtaE binds to both LuxR1 and PvdS.** (**A**) Direct interaction between LtaE-FLAG and TF-LuxR1-His. TF stands for Trigger Factor, a ribosome-associated chaperone from *E. coli*. (**B**) TF-LuxR1-His bound to GST-LtaE with moderate affinity (*K*_d_, 510 nM). (**C**) Co-localization of LtaE and LuxR1 in *E. coli*. Scale bar 10 μm. (**D**) Direct interaction between LtaE-FLAG and TF-PvdS-His. (**E**) TF-PvdS-His bound to GST-LtaE with moderate affinity (*K*_d_, 300 nM). (**F**) Co-localization of LtaE and PvdS in *E. coli*. Scale bar 10 μm.

To confirm that LtaE binds to LuxR1 and PvdS, we performed a series of biochemical assays. The direct binding of LtaE was detected to the trigger-factor-LuxR1 fusion protein, TF-LuxR1 (Fig. 3A). The microscale thermophoresis (MST) experiment revealed a moderate-to-strong affinity, *K*_d_, 0.51 μmol/L (μM) between the LuxR1-His and GST-LtaE fusions (Fig. 3B, Fig. S11A and B). Further evidence of LuxR1-LtaE interactions was obtained in the *E. coli* co-localization assays. LtaE exhibited a polar localization pattern (Fig. 3C), whereas LuxR1 was distributed throughout the cell (Fig. 3C). When LtaE and LuxR1 were coexpressed, they were colocalized at the pole (Fig. 3C). To test for the specificity of the LtaE-LuxR1 interactions, we picked another representative of the LuxR family of transcription factors from *P. protegens*, GacA, as well as an unrelated transcription factor, the phosphate regulator PhoB. None of these two transcription factors interacted with LtaE in the B2H assay, which verifies specificity of the LuxR1-LtaE interactions (Fig. S12A and B).

A similar approach confirmed specificity of the PvdS-LtaE interactions. Briefly, the pull-down assay demonstrated the binding of LtaE-FLAG to TF-PvdS (Fig. 3D). The MST experiment revealed a moderate-to-strong affinity between TF-PvdS and GST-LtaE, the dissociation constant, *K*_d_, 0.30 μM (Fig. 3E). Co-expression in *E. coli* resulted in the polar colocalization of LtaE with PvdS (Fig. 3F). The B2H assay showed no interactions between LtaE and three *P. protegens* sigma factors previously constructed in the laboratory, namely FecI, RpoS, and FliA (Fig. S13A and B).

### LtaE impairs the formation of the RNAP complexes with LuxR1 and PvdS

**Figure 4:**
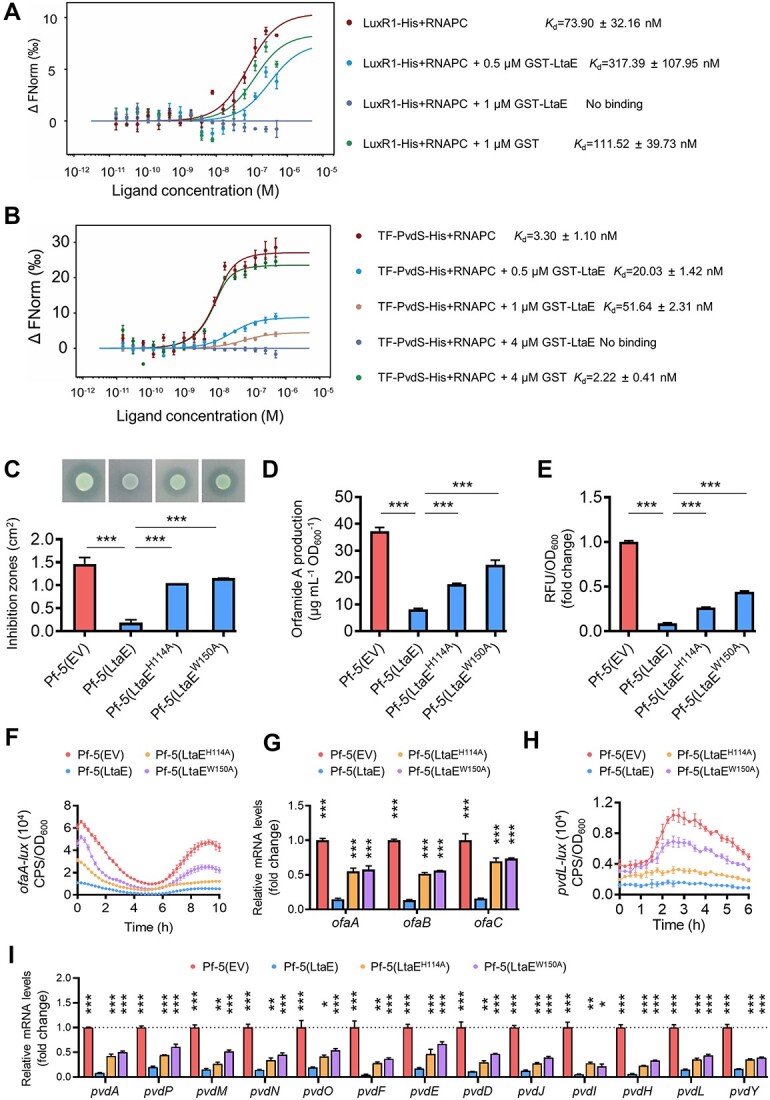
**LtaE binding dissociates the LuxR1/PvdS-RNAP complex, causing a decrease in the synthesis of orfamide A and pyoverdine in *P. protegens*.** (**A**) LuxR1 and RNAP core enzyme (RNAPC) interactions and impact of LtaE determined by MST. (**B**) PvdS and RNAPC interactions and impact of LtaE determined by MST. (**C**) *Pseudomonas protegens* Pf-5 expressing *ltaE* significantly decreased its antibacterial activity against the growth of *L. enzymogenes* OH11. LtaE^H114A^ and LtaE^W150A^ are two reported LtaE variants whose expression in *P. protegens* Pf-5 could not trigger activated antifungal effects on PDA plates [23]. (**D**) HPLC-based quantification of orfamide A yield produced by *P. protegens* Pf-5 expressing *ltaE* and two point-mutated variant genes. (**E**) Fluorescence-based quantification of pyoverdine production (fold change) in *P. protegens* Pf-5 expressing *ltaE* and two point-mutated variant genes. (**F**) Monitoring of *ofaA*-*lux* activity in *P. protegens* Pf-5 expressing *ltaE* and its two point-mutated variant genes in liquid KB. EV stands for an empty vector. (**G**) Relative mRNA expression (fold change) of the *ofaABC* operon genes analyzed by qRT-PCR. (**H**) Monitoring of *pvdL*-*lux* activity in *P. protegens* Pf-5 expressing *ltaE* and its two point-mutated variant genes in liquid KB. EV stands for an empty vector. (**I**) Relative mRNA expression (fold change) of the 13 pyoverdine operon genes analyzed by qRT-PCR. In panels (C), (D), (E), (G), and (I), results were expressed as mean ± SD from three independent biological replicates (*n* = 3). One-way ANOVA with Tukey’s multiple-comparison test was conducted. ^*^^*^^*^P < 0.001; ^*^^*^P < 0.01; ^*^P < 0.05.

We intended to elucidate the mechanisms by which LtaE interactions with LuxR1 and PvdS inhibit transcription of the *ofa* and *pvd* operons. Our initial hypothesis was that LtaE binding inhibits the ability of LuxR1 to bind DNA. However, the addition of GST-LtaE did not impair the binding of LuxR1-His to the *ofaA* DNA fragment in the EMSA (Fig. S14). We therefore explored an alternative hypothesis, i.e., that LtaE binding affects the interaction between LuxR1 and RNA polymerase (RNAP). Using MST, we observed that LuxR1-His interacts with the RNA polymerase core enzyme (RNAPC) with moderate binding affinity, *K*_d_, 73.9 nmol/L (nM) (Fig. 4A). GST-LtaE alone did not interact with RNAPC (Fig. S15A). The addition of 0.5 μM GST-LtaE to the mixture of LuxR1-His and RNAPC reduced the LuxR1-His-RNAPC binding by several-fold, from the original *K*_d_, 73.90 to 317.39 nM (Fig. 4A), whereas the addition of 1 μM completely impaired this interaction. A negative control, 1 μM GST, had only a minor effect, i.e., *K*_d_, 111.52 nM **(**Fig. 4A). These results suggest that LtaE impairs LuxR1 interaction with the RNAPC leading to the downregulation of the *ofaABC* operon transcription and decreased orfamide A production.

We investigated whether LtaE binding interferes with the formation of the PvdS-RNAPC. As expected, the MST assay revealed that the TF-PvdS and RNAPC interact with a strong affinity, *K*_d_, 3.30 nM (Fig. 4B), whereas TF-His, used as a negative control, did not bind RNAPC (Fig. S15B). The addition of 0.5 and 1 μM GST-LtaE reduced the TF-PvdS-RNAPC binding affinity by several-fold, from the original *K*_d_, 3.30 nM to 20.03 nM and 51.64 nM, respectively. The addition of higher amounts of GST-LtaE, 4 μM, resulted in no detectable binding, whereas 4 μM GST had only a slight impact (Fig. 4B). These findings indicate that LtaE downregulates pyoverdine operon transcription and pyoverdine production by interfering with the PvdS-RNAP complex formation.

### LtaE undermines the antibacterial defense systems of its competitors in the mixed cultures

Because LtaE interactions with LuxR1 and PvdS almost completely sabotaged the antibacterial activity of *P. protegens*, we predicted that expression of LtaE in *P. protegens* will result in the same outcome. This was indeed what was observed (Fig. 4C). In contrast, two LtaE mutants, LtaE^H114A^ and LtaE^W150A^, incapable of inducing the antifungal effect on the PDA media [23], failed to abolish the antibacterial activities of *P. protegens* (Fig. 4C). This result suggests that the same residues are important for LtaE interactions with different *P. protegens* transcription factors. To further probe the specificity of LtaE as a saboteur of the antibacterial activity, we expressed in *P. protegens* four additional *L. enzymogenes* T4SS effectors. None of the four impaired the antibacterial activity of *P. protegens***(**Fig. S16a–C).

Consistent with our expectations, the expression of *ltaE* significantly impaired the production of orfamide A and pyoverdine in *P. protegens* (Figs 4D, E, and S16D). Furthermore, our observations showed that LtaE markedly reduced transcription of the *ofa* and *pvd* operons, which was monitored by the *ofaA*-*lux* and *pvdL*-*lux* fusions and qRT-PCR. The H114A and W150A mutations significantly alleviated the transcriptional repression of LtaE on the expression of the *ofa* and *pvd* operons in *P. protegens* (Fig. 4F–I). These findings are consistent with the observed alleviation of LtaE repression on the production of orfamide A and pyoverdine in *P. protegens* for these two mutations (Fig. 4D–E).

**Figure 5:**
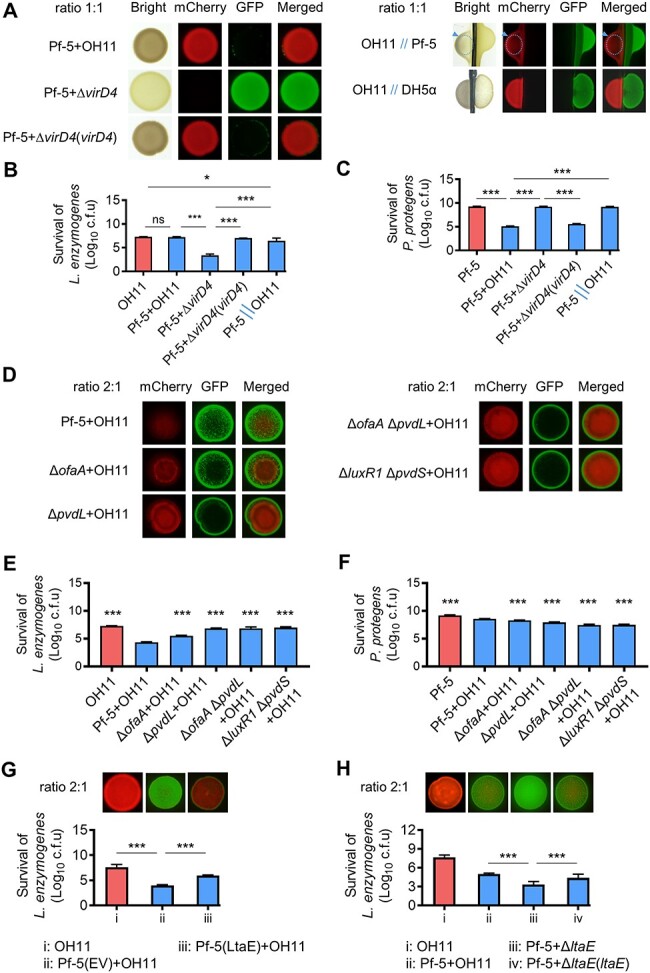
***Lysobacter** **enzymogenes*** **uses LtaE to disrupt the defense systems of *P. protegens* by blocking orfamide A and pyoverdine biosynthesis.** (**A**) Killing of *P. protegens* Pf-5 by *L. enzymogenes* OH11 using T4SS via cell-to-cell contacts. Strains of Pf-5 and OH11 or their derivatives were mixed together at a 1:1 ratio and cultivated on KB agar plates. Strain separation indicated by double slashes “//” was conducted through a 0.22 μm filter. The dashed lines and arrows indicate the damaged OH11 cells. *Pseudomonas protegens* and *E. coli* strains were labeled by GFP, and *L. enzymogenes* strains were labeled by mCherry. Pf-5, the wild-type strain of *P. protegens*; OH11, wild type of *L. enzymogenes*; Δ*virD4*, a T4SS-deficient mutant. Δ*virD4*(*virD4*), a complemented strain via the *virD4* knock-in at chromosome; DH5α, the wild-type strain of *E. coli*. (**B–C**) Quantification of *L. enzymogenes* (**B**) and *P. protegens* (**C**) living cell numbers in mixed colonies corresponding to the panel **A**. (**D**) Involvement of orfamide A and pyoverdine on the defense of *P. protegens* to *L. enzymogenes* under cell-to-cell contact conditions. *Pseudomonas protegens* strains were labeled by GFP, and *L. enzymogenes* strains were labeled by mCherry. Strains were mixed at an improved 2:1 ratio and cultivated on KB plates. Δ*ofaA*, an orfamide A-deficient mutant; Δ*pvdL*, a pyoverdine-deficient mutant; Δ*ofaA* Δ*pvdL*, an *ofaA* and *pvdL* double mutant; Δ*luxR1* Δ*pvdS*, a *luxR1* and *pvdS* double mutant. (**E–F**) Quantification of *L. enzymogenes* (**E**) and *P. protegens* (**F**) living cell numbers in mixed colonies corresponding to the panel **D**. (**G**) *Lysobacter enzymogenes* OH11 exhibited enhanced survival (living numbers) when co-cultured with *P. protegens* Pf-5 expressing *ltaE.* EV stands for empty vector. Strains of Pf-5 and OH11 were mixed together at a 2:1 ratio and cultivated on KB agar plates. (**H**) Knockout of *ltaE* in *L. enzymogenes* OH11 weakened the survival (living numbers) of Δ*ltaE* in mixed colonies with *P. protegens* Pf-5. Δ*ltaE*, an in-frame deletion mutant of *ltaE* in strain OH11. Δ*ltaE*(*ltaE*), a complemented strain via the *ltaE* knock-in at chromosome. In panels **B**, **C**, **E**, **F**, **G**, and **H**, results were expressed as mean ± SD from three independent biological replicates (*n* = 3). One-way ANOVA with Tukey’s multiple-comparison test was employed. ^*^^*^^*^P < 0.001; *P < 0.05; ns, not significant. Representative fluorescence images of three independent experiments are shown.

In contact-dependent interspecies competition experiments conducted on KB agar plates, cocultivation of *L. enzymogenes* and *P. protegens* at a 1:1 ratio led to the inhibition of *P. protegens* growth by *L. enzymogenes*. This antagonistic interaction was found to depend on the direct cell-to-cell contact and on the existence of the *L. enzymogenes* T4SS (Fig. 5A–C). In contrast to co-cultures with the wild-type *P. protegens* strain, Pf-5, *L. enzymogenes* demonstrated enhanced survival when coincubated with two single mutants, Δ*ofaA* and Δ*pvdL*_,_ and especially the double mutants, Δ*ofaA* Δ*pvdL* and Δ*luxR1* Δ*pvdS* (Fig. 5D–E). The viability of all the Pf-5 derived mutants was diminished when co-cultured with the wild-type OH11 of *L. enzymogenes* compared to the wild-type Pf-5 (Fig. 5F). Further validation was achieved through the examination of mixed colonies, revealing higher percentage of *L. enzymogenes* in the presence of *P. protegens* harboring *ltaE*, with an increase of 49.9% (Fig. 5G), but not the inactive *ltaE* mutant allele (Fig. 5H). Collectively, our data substantiate the notion that the presence of LtaE in *P. protegens* enables *L. enzymogenes* to evade competitor defenses mediated by orfamide A and pyoverdine.

### LtaE and its homologs are likely active in multiple *Pseudomonas* species

**Figure 6:**
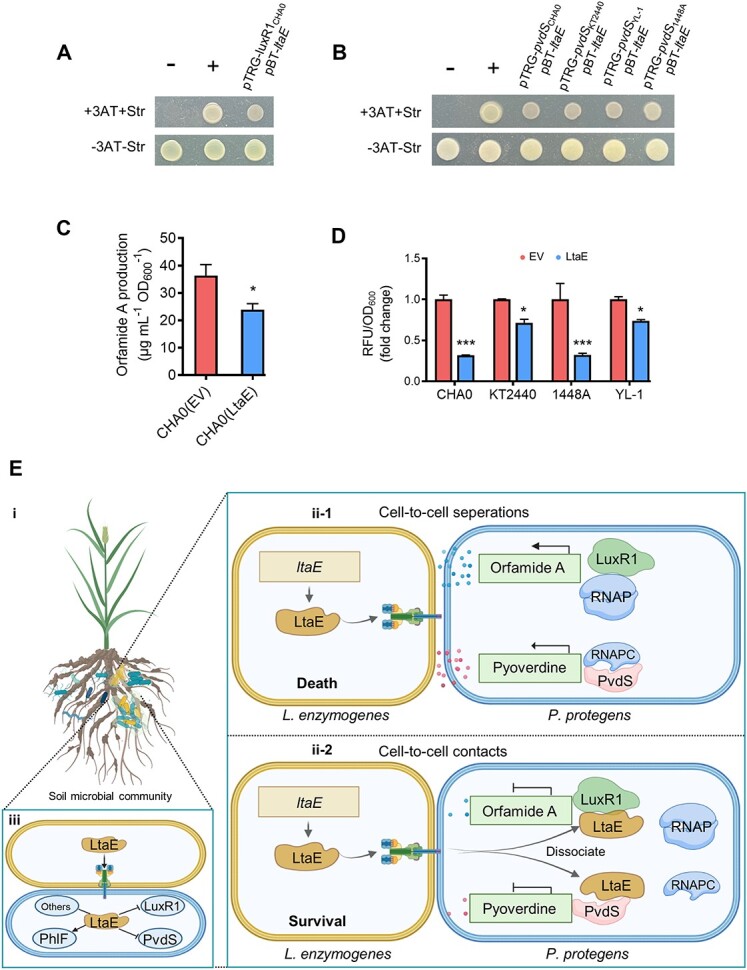
**LtaE decreases the synthesis of orfamide A and pyoverdine in multiple *Pseudomonas* species.** (**A–B**) Bacterial two-hybrid (B2H) assays show the interaction between LtaE and the LuxR1 homolog from *P. protegens* CHA0 (**A**) and the interactions between LtaE and the PvdS homologs from *P. protegens* CHA0, *P. chlororaphis* YL-1, *P. syringae* 1448A, and *P. putida* KT2440 (**B**). (**C**) HPLC-based quantification of orfamide A yield produced by *P. protegens* CHA0 expressing *ltaE*. ^*^P < 0.05, unpaired two-tailed Welch’s *t*-test was employed. Results were expressed as mean ± SD from three independent biological replicates (*n* = 3). (**D**) Quantification of pyoverdine yield (fold changes) in four *Pseudomonas* strains expressing *ltaE*. CHA0, *P. protegens*; YL-1, *P. chlororaphis*; 1448A, *P. syringae*; KT2440, *P. putida*. Two-way ANOVA with Sidak’s multiple-comparison test was employed. ^*^^*^^*^P < 0.001; ^*^P < 0.05. Results were expressed as mean ± SD from three independent biological replicates (*n* = 3). (**E**) A proposed model by BioRender illustrates the manner in which LtaE undermines multiple defense systems in *P. protegens*, a competing bacterium. *Lysobacter enzymogenes* and *P. protegens* are integral members of the ubiquitous soil microbial community (i). They co-reside in this environment, with their cells either separated or in direct contact. In instances of cell-to-cell separations (ii-1), *P. protegens* produces two diffusible antibacterial metabolites—orfamide A and pyoverdine—at elevated levels, effectively inhibiting the growth of *L. enzymogenes*. Through cell-to-cell contacts (ii-2), *L. enzymogenes* injects *LtaE* via the T4SS into the cytoplasm of *P. protegens*. Within this cytoplasmic environment, LtaE recognizes multiple protein targets, with three of them (PhlF, LuxR1, and PvdS) experimentally validated (iii). LtaE specifically binds to the pathway-associated transcriptional factor LuxR1 and the sigma factor PvdS (ii-2), which are responsible for the respective biosynthesis of orfamide A and pyoverdine. This interaction enables LtaE to hijack the assembly of the transcription complex formed by LuxR1/PvdS with the RNA polymerase core enzyme, resulting in the downregulation of the orfamide A and pyoverdine biosynthetic operon expression. Consequently, the defense systems co-mediated by the antibacterial orfamide A and pyoverdine are sabotaged by LtaE, presenting a compelling case study that underscores the significance of LtaE in recognizing multiple protein targets in prokaryotic cells.

To understand the scope of LtaE activity, we tested its interactions with the LuxR1 and PvdS homologs (Table S3) from selected *Pseudomonas* species outside *P. protegens* Pf-5. LtaE was found to interact with the LuxR1 homolog from *P. protegens* CHA0 (Fig. 6A and Fig. S17) and with PvdS homologs from *P. chlororaphis* YL-1, *P. syringae* 1448A, and *Pseudomonas putida* KT2440 (Fig. 6B and Fig. S18). Consistent with the results of protein–protein interactions, LtaE negatively impacted orfamide A production in *P. protegens* CHA0 (Fig. 6C and Fig. S19) and hindered pyoverdine production in *P. chlororaphis* YL-1, *P. syringae* 1448A, *P. putida* KT2440, and *P. protegens* CHA0 (Fig. 6D). Moreover, LtaE expression in *Pseudomonas* strain N15-1, the strain isolated from the pepper rhizosphere, the same ecological niche from which *L. enzymogenes* OH11 was isolated [35], pyoverdine production was impaired (Figs S20–S21). These results show that LtaE can disable antibacterial defenses in a variety of competing pseudomonads.

We extended our experiments to encompass multiple *Lysobacter* and *Pseudomonas* species to enhance the generalizability of our findings. By conducting LtaE sequence comparison, we identified Lg3853 protein from *Lysobacter gummosus* OH17, Lb2713 protein from *Lysobacter brunescens*, and GLE5041 protein from *L. enzymogenes* C3 as LtaE homologs with (Table S4 and Fig. S22). B2H assays revealed that these three LtaE homologs, similar to LtaE, interacted with the PvdS and its homologs from *P. protegens* Pf-5 and CHA0, *P. chlororaphis* YL-1, *P. syringae* 1448A, and *P. putida* KT2440 (Fig. S23a–E). Additionally, fluorescence quantification tests indicated that, like LtaE, all tested LtaE homologs also inhibited pyoverdine production when their protein genes were expressed in *P. protegens* Pf-5 and CHA0, *P. chlororaphis* YL-1, *P. syringae* 1448A, and *P. putida* KT2440 (Fig. S24a–E). Furthermore, we observed that LuxR1 from *P. protegens* Pf-5 and CHA0 are identical at the amino acid sequence level (Fig. S17). Whereas all three LtaE homologs interacted with LuxR1 from both strains (Pf-5 and CHA0) as shown by B2H (Fig. S25a–B), HPLC experiments revealed that all three LtaE homologs inhibited orfamide A production only in *P. protegens* Pf-5, but not in CHA0 (Fig. S26a–D). Although the specific reasons for this unexpected phenomenon are unclear, it is possible that *P. protegens* CHA0 might counteract the action of LtaE homologs by encoding an unknown, strain-specific factor that obstructs the direct interactions between its LuxR1 and the LtaE homologs tested in this study.

## Discussions

A single effector injected by a pathogen into a eukaryotic host can target multiple host defense systems [36–38]. Our work shows that this phenomenon extends to interspecies interactions in competing bacteria. We discovered that a single T4SS effector from *L. enzymogenes*, LtaE, attacks multiple targets in the competing soil bacterium, *P. protegens*. Binding to LuxR1 and PvdS enables this bacterial effector to block synthesis of the antimicrobials, orfamide A and pyoverdine, that are toxic to *L. enzymogenes* (Fig. 6E). Given that LtaE recognizes several LuxR1 and PvdS homologs, it appears that LtaE can subdue antibacterial systems not only in *P. protegens* but also in a variety of other pseudomonads living in the soil and on plant matter.

Soil bacteria produce and diverse metabolites with a range of antimicrobial activities against bacterial and fungal pathogens [39]. These metabolites, due to their diffusible nature, act independently of direct cell-to-cell contacts, functioning as “long-range” antimicrobial weapons [40, 41]. Recent studies have uncovered the use of “short-range” antimicrobial weapons by soil bacteria that target their competitors [20, 42–46]. These short-range weapons operate via cell-to-cell contacts and translocate multiple protein effectors, distinct from antimicrobial metabolites. The T4SS and type VI secretion system (T6SS) represent such short-range antimicrobial weapons, often used by the proteobacteria to gain advantages in mixed microbial communities [20, 42–46]. The range of functions of these short-range weapons appears to be broad. The primary role of T4SS is believed to be translocating lethal effectors that target competitor’s cell wall and membranes [42, 44]. Our earlier studies revealed that T4SS effectors may have different roles. Specifically, *L. enzymogenes* T4SS effector, LqqE1, is involved in quorum quenching, which presumably blocks the ability of the competitor bacteria from mounting a quorum sensing-dependent attack. Following injection, LqqE1 forms a protein complex with the quorum sensing molecule synthase, PcoI, thus inhibiting its activity [19]. Here, we demonstrated that a different T4SS effector, LtaE, suppresses the production of two long-range antibacterial weapons, orfamide A and pyoverdine, in a competing bacterium by binding to two pathway-specific transcription regulators, LuxR1 and PvdS. Mechanistically, LtaE disrupts the formation of the transcriptional complexes between these regulators and the RNAPC.

Whereas transcriptional “reprogramming” of the competitor’s antibacterial weaponry by a T4SS effector has been described here, our earlier study showed that LtaE can “reprogram” transcription of an antifungal metabolite, under different conditions. Specifically, on the PDA agar media, LtaE binds to PhlF, the transcriptional repressor of biosynthesis of the antifungal agent, 2,4-DAPG [23]. It is possible that under the nutrient-depleted conditions mimicked by the PDA media, LtaE injection serves to coerce *P. protegens* to cooperate with *L. enzymogenes* in fight with competing fungi. On the more nutritious media like KB, *L. enzymogenes* turns from a collaborator to a competitor that sabotages production of the *P. protegens* long-range antibacterial weapons, orfamide A and pyoverdine. Therefore, a single T4SS effector capable of recognizing multiple targets in a competing bacterium may control its behavior based on the environmental conditions. Moreover, the conservative inhibition of pyoverdine production in *Pseudomonas* pathogens such as *P. syringae* 1448A by LtaE and its homologs from various biocontrol *Lysobacter* species suggests the potential for these biocontrol *Lysobacter* to protect plants from pathogen infection. This protection may be achieved by enhancing their killing efficiency against *Pseudomonas* pathogens through the injection of LtaE and its homologous proteins, which likely blocks the pyoverdine-mediated antibacterial weapon. In future studies, transcriptomic analysis of *Pseudomonas* strains upon expression of LtaE and its homologs could be conducted to deepen the understanding of the phenomena observed in this study.

## Supplementary Material

Wang_ISME_supplemental_file_20240627_wrae121

## Data Availability

The sequence data from the present study have been submitted to the NCBI GenBank under the following accession numbers: MW052471 (Le1519), PP932005 (Lb2713), PP932006 (Lg3853), ALN60382 (GLE5041), AAY91417 (LuxR1), AGL83956 (LuxR1_CHA0), AAY93446 (PvdS), AGL86013 (PvdS_CHA0), AAZ32988 (PvdS_1448A), WP_009050074 (PvdS_YL-1), AAN69824 (PvdS_KT2440), and PP930753 (*Pseudomonas* sp. N15-1 16s rRNA).
