# Supplementary material for: Type IV secretion system effector sabotages multiple defense systems in a competing bacterium
Source: ISME J. 2024 Jul 3;18(1):wrae121. doi: 10.1093/ismejo/wrae121 (PMC11253431; doi:10.1093/ismejo/wrae121)
Supplement: Wang_ISME_supplemental_file_20240627_wrae121 [file wang_isme_supplemental_file_20240627_wrae121.docx]

**Supplementary Information**

**Type IV secretion system effector sabotages multiple defense systems in a competing bacterium**

Bingxin Wang^1^, Fugui Xu^1^, Zeyu Zhang^1^, Danyu Shen^1^, Limin Wang^1^, Huijun Wu^1^, Qing Yan^2^, Chuanbin Cui^3^, Pingping Wang^3^, Qi Wei^4^, Xiaolong Shao^1^, Mengcen Wang^5, 6^, Guoliang Qian^1^*

This file contains 4 supplemental tables and 26 supplemental figures.

**Table S1 Bacterial strains and plasmids used in this study**

| **Strains or plasmids** | **Characteristics^a^** | **Source** |
| --- | --- | --- |
| **Plasmids** | | |
| pCold TF | Vector for IPTG-inducible gene expression, Amp^R^ | Lab stock |
| pCold TF-*luxR1* | pCold TF carrying *luxR1* coding region, Amp^R^ | This study |
| pCold TF-mCherry | pCold TF carrying mCherry coding region, Amp^R^ | This study |
| pCold TF-*pvdS* | pCold TF carrying *pvdS* coding region, Amp^R^ | This study |
| pBAD/Myc-His A | Vector for arabinose-inducible gene expression, Amp^R^ | Lab stock |
| pBADGM | Vector for arabinose-inducible gene expression, Gm^R^ | Lab stock |
| pBAD-LtaE*-*FLAG | pBAD/Myc-His A carrying LtaE*-*FLAG coding region, Amp^R^ | Lab stock |
| pBAD-GFP*-*FLAG | pBAD/Myc-His A carrying GFP*-*FLAG coding region, Amp^R^ | Lab stock |
| pBAD-GFP | pBAD/Myc-His A carrying GFP coding region, Amp^R^ | Lab stock |
| pBAD-LtaE::GFP | pBAD/Myc-His A carrying LtaE-GFP coding region, Amp^R^ | Lab stock |
| pBADGM-mCherry | pBADGM carrying mCherry coding region, Gm^R^ | Lab stock |
| pBADGM-LuxR1::mCherry | pBADGM carrying LuxR1::mCherry coding region, Gm^R^ | Lab stock |
| pBADGM-PvdS::mCherry | pBADGM carrying PvdS::mCherry coding region, Gm^R^ | Lab stock |
| pMS402 | reporter plasmid carrying a promoterless *luxCDABE* reporter, Kan^R^ | Lab stock |
| pMS402-*pvdL* | pMS402 carrying promoter of *pvdL*, Kan^R^ | Lab stock |
| pMS402-*ofaA* | pMS402 carrying promoter of *ofaA*, Kan^R^ | Lab stock |
| pVSP61 | Broad-host-range vector with a P*_lac_* promoter, Kan^R^ | Lab stock |
| pVSP61-*luxR1* | pVSP61 carrying *luxR1* coding region, Kan^R^ | This study |
| pVSP61-*pvdS* | pVSP61 carrying *pvdS* coding region, Kan^R^ | This study |
| pK18mobsacB | Suicide vector with a *sacB* gene, Kan^R^ | Lab stock |
| pK18-*pvdL* | pK18mobsacB with two flanking fragments of *pvdL*, Kan^R^ | This study |
| pK18-*pvds* | pK18mobsacB with two flanking fragments of *pvdS*, Kan^R^ | This study |
| pK18-*ofaA-cp* | pK18mobsacB containing flanking fragments of *ofaA* and its coding region, Kan^R^. | This study |
| pK18-*pvdL-cp* | pK18mobsacB containing flanking fragments of *pvdL* and its coding region, Kan^R^. | This study |
| pBBR1-MCS-5 | Broad-host-range vector with a P*_lac_* promoter, Gm^R^ | Lab stock |
| pBBR5-LtaE-FLAG | pBBR1MCS-5 carrying LtaE-FLAG coding region, Gm^R^ | Lab stock |
| pBBR-LtaE^W150A^ | pBBR1MCS-5 carrying LtaE^W150A^ coding region, Gm^R^ | Lab stock |
| pBBR-LtaE^H114A^ | pBBR1MCS-5 carrying LtaE^H114A^ coding region, Gm^R^ | Lab stock |
| **Strains** |  |  |
| XL1-Blue MRF^’^ Kan | Host for bacterial two-hybrid assay, Km^R^ | Lab stock |
| XL1-Blue MRF^’^ Kan (+) | XL1-Blue MRF^’^ Kan harbouring plasmids pBT-gacS and pTRG-gacS, Km^R^, Cm^R^, and Tet^R^ | Lab stock |
| XL1-Blue MRF^’^ Kan (-) | XL1-Blue MRF^’^ Kan harbouring plasmids pBT and pTRG, Km^R^, Cm^R^, and Tet^R^ | Lab stock |
| *Escherichia coli* Top 10 | Host strain for molecular cloning | Lab stock |
| BL21(DE3) | Host strain for protein expression | Lab stock |
| BL21(DE3) (GST-LtaE) | BL21(DE3) harbouring plasmid pGEX-6p-1-LtaE, Amp^R^ | ^1^ |
| BL21(DE3) (TF-LuxR1) | BL21(DE3) harbouring plasmid pCold TF-*luxR1*, Amp^R^ | This study |
| BL21(DE3) (TF-mcherry) | BL21(DE3) harbouring plasmid pCold TF-*luxR1*, Amp^R^ | This study |
| BL21(DE3) (LtaE-FLAG) | BL21(DE3) harbouring plasmid pBAD-LtaE*-*FLAG, Amp^R^ | ^1^ |
| BL21(DE3) (GFP-FLAG) | BL21(DE3) harbouring plasmid pBAD-GFP*-*FLAG, Amp^R^ | ^1^ |
| BL21(DE3) (TF-*pvdS*) | BL21(DE3) harbouring plasmid pCold TF-*pvdS*, Amp^R^ | This study |
| BL21(DE3) (LuxR1-His) | BL21(DE3) harbouring plasmid pBAD-LuxR1-His, Amp^R^ | This study |
| *Pseudmonas protegen* Pf-5 | Wild type, Amp^R^ | Lab stock |
| Pf-5(*ofaA*-*lux*) | Pf-5 harbouring plasmid pMS402-*ofaA*, Km^R^ | This study |
| Pf-5Δ*luxR1*(*ofaA*-*lux*) | Pf-5Δ*luxR1* harbouring plasmid pMS402-*ofaA*, Km^R^ | This study |
| Pf-5(*pvdL*-*lux*) | Pf-5 harbouring plasmid pMS402-*pvdL*, Km^R^ | This study |
| Pf-5Δ*pvdS*(*pvdL*-*lux*) | Pf-5Δ*pvdS* harbouring plasmid pMS402-*ofaA*, Km^R^ | This study |
| Δ*ofaA* | Out-of-frame deletion of *ofaA* in strain Pf-5, Amp^R^ | This study |
| Δ*ofaA*(*ofaA*) | Chromosomal complementation of *ofaA* in Pf-5Δ*ofaA*, Amp^R^ | This study |
| Δ*luxR1* | In-frame deletion of *PFL2143* in strain Pf-5, Amp^R^ | This study |
| Δ*luxR1*(EV) | Δ*luxR1* harbouring plasmid pVSP61, Amp^R^, Kan^R^ | This study |
| Δ*luxR1*(*luxR1*) | Δ*luxR1* harbouring plasmid pVSP61-*luxR1*, Amp^R^, Kan^R^ | This study |
| Δ*pvdS*(EV) | Δ*pvdS* harbouring plasmid pVSP61, Amp^R^, Kan^R^ | This study |
| Δ*pvdS*(*pvdS*) | Δ*pvdS* harbouring plasmid pVSP61-*pvdS*, Amp^R^, Kan^R^ | This study |
| Δ*pvdL* | Out-of-frame deletion of *pvdL* in strain Pf-5, Amp^R^ | This study |
| Δ*pvdL*(*pvdL*) | Chromosomal complementation of *pvdL* in Δ*pvdL*, Amp^R^ | This study |
| Δ*ofaA* Δ*pvdL* | Out-of-frame deletion of *pvdL* in strain Δ*ofaA*, Amp^R^ | This study |
| Δ7 | In-frame deletion of *phlD*, *pltB, ofaA, prnA, hcnABC, PFL4656, and rzxB* in strain Pf-5, Amp^R^ | ^1^ |
| Δ8 | In-frame deletion of *pvdL* in strain Δ7, Amp^R^ | This study |
| Δ8(*pvdL*) | Chromosomal complementation of *pvdL* in Δ8, Amp^R^ | This study |
| Pf-5(EV) | Pf-5 harbouring plasmid pBBR1MCS-5, Amp^R^, Gm^R^ | ^1^ |
| Pf-5(LtaE) | Pf-5 harbouring plasmid pBBR-Le1519-FLAG, Amp^R^, Gm^R^ | ^1^ |
| Pf-5(LtaE^H114A^) | Pf-5 harbouring plasmid pBBR-LtaE^H114A^, Amp^R^, Gm^R^ | ^1^ |
| Pf-5(LtaE^W150A^) | Pf-5 harbouring plasmid pBBR-LtaE^W150A^, Amp^R^, Gm^R^ | ^1^ |
| Pf-5(LtaE+*ofaA*-*lux*) | Pf-5(LtaE) harbouring plasmid pMS402-*ofaA*, Km^R^, Gm^R^ | ^1^ |
| Pf-5(EV+*ofaA*-*lux*) | Pf-5(EV) harbouring plasmid pMS402-*ofaA*, Km^R^, Gm^R^ | ^1^ |
| Pf-5(LtaE^H114A^+*ofaA*-*lux*) | Pf-5(LtaE^H114A^) harbouring plasmid pMS402-*ofaA*, Km^R^, Gm^R^ | This study |
| Pf-5(LtaE^W150A^+*ofaA*-*lux*) | Pf-5(LtaE^W150A^) harbouring plasmid pMS402-*ofaA*, Km^R^, Gm^R^ | This study |
| Pf-5(LtaE+*pvdL*-*lux*) | Pf-5(LtaE) harbouring plasmid pMS402-*pvdL*, Km^R^, Gm^R^ | This study |
| Pf-5(EV+*pvdL*-*lux*) | Pf-5(EV) harbouring plasmid pMS402-*pvdL*, Km^R^, Gm^R^ | This study |
| Pf-5(LtaE^H114A^+*pvdL*-*lux*) | Pf-5(LtaE^H114A^) harbouring plasmid pMS402-*pvdL*, Km^R^, Gm^R^ | This study |
| Pf-5(LtaE^W150A^+*pvdL*-*lux*) | Pf-5(EV) harbouring plasmid pMS402-*pvdL*, Km^R^, Gm^R^ | This study |
| *Lysobacter enzymogenes* OH11 | Wild type, Km^R^ | Lab stock |
| Δ*virD4* | In-frame deletion of *virD4* in strain OH11, Km^R^ | Lab stock |
| Δ*virD4*(*virD4*) | Chromosomal complementation of *virD4* in *Le*Δ*virD4*, Km^R^ | This study |
| Δ*ltaE* | In-frame deletion of *ltaE* in strain OH11, Km^R^ | This study |
| Δ*ltaE*(*ltaE*) | Chromosomal complementation of *ltaE* in Δ*ltaE*, Km^R^ | This study |
| *Pseudomonas chlororaphis* YL-1 | Wild type | Lab stock |
| YL-1(EV) | YL-1 harbouring plasmid pBBR1MCS-5, Gm^R^ | This study |
| YL-1(LtaE) | YL-1 harbouring plasmid pBBR-LtaE-FLAG, Gm^R^ | This study |
| 1448A(EV) | 1448A harbouring plasmid pBBR1MCS-5, Gm^R^ | This study |
| 1448A(LtaE) | 1448A harbouring plasmid pBBR-LtaE-FLAG, Gm^R^ | This study |
| *Pseudomonas putida* KT2440 | Wild type | Lab stock |
| KT2440(EV) | KT2440 harbouring plasmid pBBR1MCS-5, Gm^R^ | This study |
| KT2440(LtaE) | KT2440 harbouring plasmid pBBR-LtaE-FLAG, Gm^R^ | This study |

^a^ Km^R^, Amp^R^, CM^R^, Tet^R^, and Gm^R^ indicate resistance to Kanamycin, Ampicillin, chloramphenicol, tetracycline, and Gentamicin respectively.

**Table S2. Primers used in this study**

| **Primers** | **5’-3’ sequence^a^** | **Function** |
| --- | --- | --- |
| pcold-*pvds*-F | ggtatcgaaggtaggcatatgACGGAACAAGTATCCACAAG | To generate  pCold TF-*pvdS* |
| pcold-*pvds*-R | gacaagcttgaattcggatccTTAGCGACGGGCGAAGGTAT |  |
| pcold-*luxR1*-F | ggtatcgaaggtaggcatatgAGTCTGACCAGCAGTATTGC | To generate  pCold TF-*luxR1* |
| pcold-*luxR1*-R | gacaagcttgaattcggatccGGCGCCGACCATCCACTTGG |  |
| pcold-mCherry-F | ggtatcgaaggtaggcatatgGTGAGCAAGGGCGAGGAGG | To generate  pCold TF-mCherry |
| pcold-mCherry-R | gacaagcttgaattcggatccCTTGTACAGCTCGTCCATG |  |
| pBAD-LuxR1-F | tcgagatctgcagctggtaccAGTCTGACCAGCAGTATTGC | To generate  pBAD-LuxR1-His |
| pBAD-LuxR1-R | tttttgttcgggcccaagcttGGCGCCGACCATCCACTTGG |  |
| pVSP61-*luxR1*-F | gaccatgattacgccaagcttgAGTCTGACCAGCAGTATTGC | To generate  pVSP61-*luxR1* |
| pVSP61-*luxR1*-R | aaaacgacggccagtgaattcTCAATGATGATGATGATGATGGGCGCCGACCA |  |
| pVSP61-*pvdS*-F | gaccatgattacgccaagcttgACGGAACAAGTATCCACAAG | To generate  pVSP61-*pvdS* |
| pVSP61-*pvdS*-R | aaaacgacggccagtgaattcTCAATGATGATGATGATGATGGTCGACGGCGC |  |
| pk18-*pvdL*-U1 | ctatgacatgattacgaattcACTTCCACCTCGCGCTCG | To generate  pK18-*pvdL* |
| pk18-*pvdL*-U2 | gtaTGGAAGCGATCAGCGAATACG |  |
| pk18-*pvdL*-D1 | ttcgctgatcgcttccaTACCGCGCGATCGCCGAA |  |
| pk18-*pvdL*-D2 | caggtcgactctagaggatccATCGGCAGCCGGTAATGC |  |
| pk18-*pvds*-U1 | ctatgacatgattacgaattcATGGAAATCAGGCGCTCCTG | To generate  pK18-*pvds* |
| pk18-*pvds*-U2 | gaaGAGTAACGGTGAATCGCACCTG |  |
| pk18-*pvds*-D1 | gcgattcaccgttactcTTCATGATTCGCGATGCCC |  |
| pk18-*pvds*-D2 | caggtcgactctagaggatccCACGGAGCAGTTGCTGGTTT |  |
| pk18-*ofaA*-U1 | ctatgacatgattacgaattcCCAGGTTCGCAATACTGCTGG | To generate  pK18-*ofaA* |
| pk18-*ofaA*-U2 | tcatgcgttcgtgcaCAGTTGGTCGAGCCAGATATCG |  |
| pk18-*ofaA*-D1 | aactgTGCACGAACGCATGAAGGC |  |
| pk18-*ofaA*-D2 | caggtcgactctagaggatccCGGGCGTGAATCCGCTGT |  |
| pk18-*pvdL*-U1 | ctatgacatgattacgaattcACTTCCACCTCGCGCTCG | To generate  pK18-*pvdL-cp* |
| pk18-*pvdL*-D2 | caggtcgactctagaggatccATCGGCAGCCGGTAATGC |  |
| pk18-*ofaA*-U1 | ctatgacatgattacgaattcCCAGGTTCGCAATACTGCTGG | To generate  pK18-*ofaA-cp* |
| pk18-*ofaA*-D2 | caggtcgactctagaggatccCGGGCGTGAATCCGCTGT |  |
| *rrsa*-F | ACCTGGACTGATACTGACACTGAG | To test the transcription of 16s rRNA |
| *rrsa*-R | AGGCGGTCAACTTAATGCGTTAG |  |
| *ofaA*-F | TGCGCGATGTACTGAAACAG | To test the transcription of *ofaA* |
| *ofaA*-R | TTGGAGACTTTCACCGACGA |  |
| *ofaB*-F | CCCTGGACCTGCTGGTGAGC | To test the transcription of *ofaB* |
| *ofaB*-R | GCGGTCCAGGTCGACCCTTC |  |
| *ofaC*-F | GATGAACGCATCAGCACCTT | To test the transcription of *ofaC* |
| *ofaC*-R | TAGATGCGGGTATTGGCGAT |  |
| *pvdA*-F | TTGGTGTTCTGGTACTCGCT | To test the transcription of *pvdA* |
| *pvdA*-R | CGACCTCAACGACAGTTTCC |  |
| *pvdP*-F | TGGCGTCGAGAAACTCGATA | To test the transcription of *pvdP* |
| *pvdP*-R | GCGTACTACACCCAGAAGGA |  |
| *pvdM*-F | CATCCTCGATGAGGAACCCA | To test the transcription of *pvdM* |
| *pvdM*-R | CAGTTTCTGCACCTGTTCCC |  |
| *pvdN*-F | CCGTCGGACATTCCTCAAAC | To test the transcription of *pvdN* |
| *pvdN*-R | TTCCAGGTGTACTGGGTGTC |  |
| *pvdO*-F | CCAGCAAACCACGCTATGAA | To test the transcription of *pvdO* |
| *pvdO*-R | GGTGATCTGGTACTGGCCTT |  |
| *pvdF*-F | CTCATAGGGCGATTCGATGC | To test the transcription of *pvdF* |
| *pvdF*-R | GTCAACGACTTGCTGCTGAG |  |
| *pvdE*-F | TACCTGAACCGCCTGGAAAT | To test the transcription of *pvdE* |
| *pvdE*-R | GTGGCTGATCACGATGATGG |  |
| *pvdD*-F | GACAAGGCGCTCAAGTCATT | To test the transcription of *pvdD* |
| *pvdD*-R | AGATCCAGACGCAGGAGATG |  |
| *pvdJ*-F | CTTCACCTGATGGTCGATGC | To test the transcription of *pvdJ* |
| *pvdJ*-R | AGCAACACCCAGAGCTACAT |  |
| *pvdI*-F | AATGCATGGGCAGCATGTAG | To test the transcription of *pvdI* |
| *pvdI*-R | AAGATCCGCGGTTACCGTAT |  |
| *pvdH*-F | GTCCTGGACGAACTGATCCT | To test the transcription of *pvdH* |
| *pvdH*-R | CAATGCCCGCAGTTATCCAC |  |
| *pvdL*-F | CAGCTCGACGGGCAGATGAG | To test the transcription of *pvdL* |
| *pvdL*-R | ACTGCCGGCTTTGCTGGAAT |  |
| *pvdY*-F | GCTGCACGTTTATGCAACAG | To test the transcription of *pvdY* |
| *pvdY*-R | CTCACGGGAATCGATGAGGT |  |
| pTRG-*pvds*-F | aaaccagaggcggccggatccATGACGGAACAAGTATCCACAAG | To generate  pTRG-*pvdS* |
| pTRG-*pvds*-E | gcgccagctcagactgaattcTCAGCGACGGGCGAAGGT |  |
| *fecI*-pTRG-F | aaaccagaggcggccggatccTCCCACACCGCTACCGTCG | To generate  pTRG-*fecI* |
| *fecI*-pTRG-R | ttaattaattaattactcgagTCACAGGGCGAAGTAGCAGC |  |
| pTRG-*fleQ*-F | aaaccagaggcggccggatccTGGCGTGAAACCAAAATTCTG | To generate  pTRG-*fleQ* |
| pTRG-*fleQ*-R | gcgccagctcagactgaattcTCAATCATCCGCCTGTTCATC |  |
| pTRG-*phoB*-F | aaaccagaggcggccggatccGTTGGCAGAAGCATTCTGATC | To generate  pTRG-*phoB* |
| pTRG-*phoB*-R | gcgccagctcagactgaattcTCAGGCCTTGGTGGAAAAACG |  |
| *rpoS*-pTRG-F | aaaccagaggcggccggatccGCTCTCAGTAAAGAAGTGCCGG | To generate  pTRG-*rpoS* |
| *rpoS*-pTRG-R | ttaattaattaattactcgagCTACTGAAACAGCGACTCACTGGA |  |
| *fliA*-pTRG-F | aaaccagaggcggccggatccACTGCCAGTGGCTACAACCTCT | To generate  pTRG-*fliA* |
| *fliA*-pTRG-R | ttaattaattaattactcgagTCAGCGCGCTCGCCACTC |  |
| *gacA*-pTRG-F | aaaccagaggcggccggatccATAAGGGTGCTAGTAGTCGATGACC | To generate  pTRG-*gacA* |
| *gacA*-pTRG-R | ttaattaattaattactcgagTCAGAGGCTGGCATCAACCA |  |
| pTRG-2785-F | aaaccagaggcggccggatccAAGGCGCTAGGCGTTGTCA | To generate  pTRG-2785 |
| pTRG-2785-R | gcgccagctcagactgaattcTCATGATGTCTGGCTCTTGTCTTG |  |
| pTRG-*luxR1*-F | aaaccagaggcggccggatccATGAGTCTGACCAGCAGTAT | To generate  pTRG-*luxR1* |
| pTRG-*luxR1*-R | ttaattaattaattactcgagTCAGGCGCCGACCATCCACT |  |
| pTRG-3497-F | aaaccagaggcggccggatccCTCAGCCCCAACGCTGCG | To generate |
| pTRG-3497-R | gcgccagctcagactgaattcTCAACGCAAGGACTTGGGC | pTRG-3497 |
| pTRG-3599-F | aaaccagaggcggccggatccGAAATTCGTCACTTTCGCTACTTC | To generate  pTRG-3599 |
| pTRG-3599-R | gcgccagctcagactgaattcTCAGGCTTTGCCGCGGTA |  |
| pTRG-3610-F | aaaccagaggcggccggatccATCGCCGCCGTGTCTTATC | To generate  pTRG-3610 |
| pTRG-3610-R | gcgccagctcagactgaattcTCAGTCATCGGCCAGGTGC |  |
| pTRG-2573-F | aaaccagaggcggccggatccACCCGCATTCTGACCATCG | To generate  pTRG-2573 |
| pTRG-2573-R | gcgccagctcagactgaattcTTAGAGGGGTTCAGCAATGACA |  |
| pTRG-2582-F | aaaccagaggcggccggatccAGCCGTGGAAAACACCCC | To generate  pTRG-2582 |
| pTRG-2582-R | gcgccagctcagactgaattcTCAGCGGCCTTCTACAGGC |  |
| pTRG-2986-F | aaaccagaggcggccggatccCCTAACTTCGCAGATCTGGA | To generate  pTRG-2986 |
| pTRG-2986-R | gcgccagctcagactgaattcCTAGCGGGTGTCCTGGGTC |  |
| pTRG-4639-F | aaaccagaggcggccggatccCGTATCCACGTCAGCTTCATC | To generate  pTRG-4639 |
| pTRG-4639-R | gcgccagctcagactgaattcTCAGGCCTTGCTGGGAATG |  |
| pTRG-3479-F | aaaccagaggcggccggatcc ATGAGCCACATCGATCTGAACC | To generate  pTRG-3479 |
| pTRG-3479-R | Gcgccagctcagactgaattc TCAAGACTGGCCGTCGATGATCG |  |
| pTRG-pvdS 1448-F | aaaccagaggcggccggatccACGGAACACGTAATCACAAGTAAGTG | To generate  pTRG-*pvdS*_1448A_ |
| pTRG-pvdS 1448-R | gcgccagctcagactgaattcTCACGCTTCAGCGTTGGC |  |
| pTRG-pvdS KT2440-F | aaaccagaggcggccggatccGCGGAACAACTATCCACAAGTAAG | To generate  pTRG-*pvdS*_KT2440_ |
| pTRG-pvdS KT2440-R | gcgccagctcagactgaattcTCAGGCCTGGCGACTGGC |  |
| pTRG-pvdS YL-1-F | aaaccagaggcggccggatccACGGAACAAGTATCCACAAG | To generate  pTRG-*pvdS*_YL-1_ |
| pTRG-pvdS YL-1-R | gcgccagctcagactgaattc TCAGTGGCGGGCGTAGGT |  |
| pBT-*ltaE*-F | tggcgcggccgcatcgaattccATGAGCGGATTGACCCAGCAA | To generate  pBT-*ltaE* |
| pBT-*ltaE*-R | aattaattaactcgaggatcc TCAGACCATCGCCCGCGCCGCC |  |
| pBT-GLE5041-F | tggcgcggccgcatcgaattcc AGCGGATTGACCCAGCAAGA | To generate  pBT-GLE5041 |
| pBT-GLE5041-R | aattaattaactcgaggatccTCAAACCATCGCCCGCGCCG |  |
| pBT-Lg3853-F | tggcgcggccgcatcgaattccAGCGGATTGAACGAACGCGA | To generate  pBT-Lg3853 |
| pBT-Lg3853-R | aattaattaactcgaggatccGGCCATGCTGCGCGCCGGAT |  |
| pBT-Lb2713-F | tggcgcggccgcatcgaattccAGCGGATTGAATCGCCAGGA | To generate  pBT-Lb2713 |
| pBT-Lb2713-R | aattaattaactcgaggatccTCACAGCACCCGCGTCGGCGC |  |

^a^ The lowercase letters indicate homologous fragments of the vector for cloning.

**Table S3 Sequence identity and similarity between PvdS and its homologs**

| PvdS homologs | Accession number | Organism | Identity (%)^a^ | Similarity (%)^a^ |
| --- | --- | --- | --- | --- |
| PvdS_CHA0 | AGL86013 | *P. protegens* | 100 | 100 |
| PvdS_YL-1 | WP_009050074 | *P. chlororaphis* | 95.1 | 98.4 |
| PvdS_1448A | AAZ32988 | *P. syringae* | 89.6 | 84.1 |
| PvdS_KT2440 | AAN69824 | *P. putida* | 80.2 | 87.9 |

^a^ The values represent the sequence identity and similarity percentages obtained using the Needleman-Wunsch algorithm.

**Table S4 Sequence identity and similarity between LtaE and its homologs**

| LtaE homologs | Accession number | Organism | Identity (%)^a^ | Similarity (%)^a^ |
| --- | --- | --- | --- | --- |
| GLE5041 | ALN60382 | *L. enzymogenes* | 97.1 | 98.3 |
| Lg3853 | PP932006 | *L. gummosus* | 50.3 | 62.0 |
| Lb2713 | PP932005 | *L. brunescens* | 45.0 | 57.9 |

^a^ The values represent the sequence identity and similarity percentages obtained using the Needleman-Wunsch algorithm.


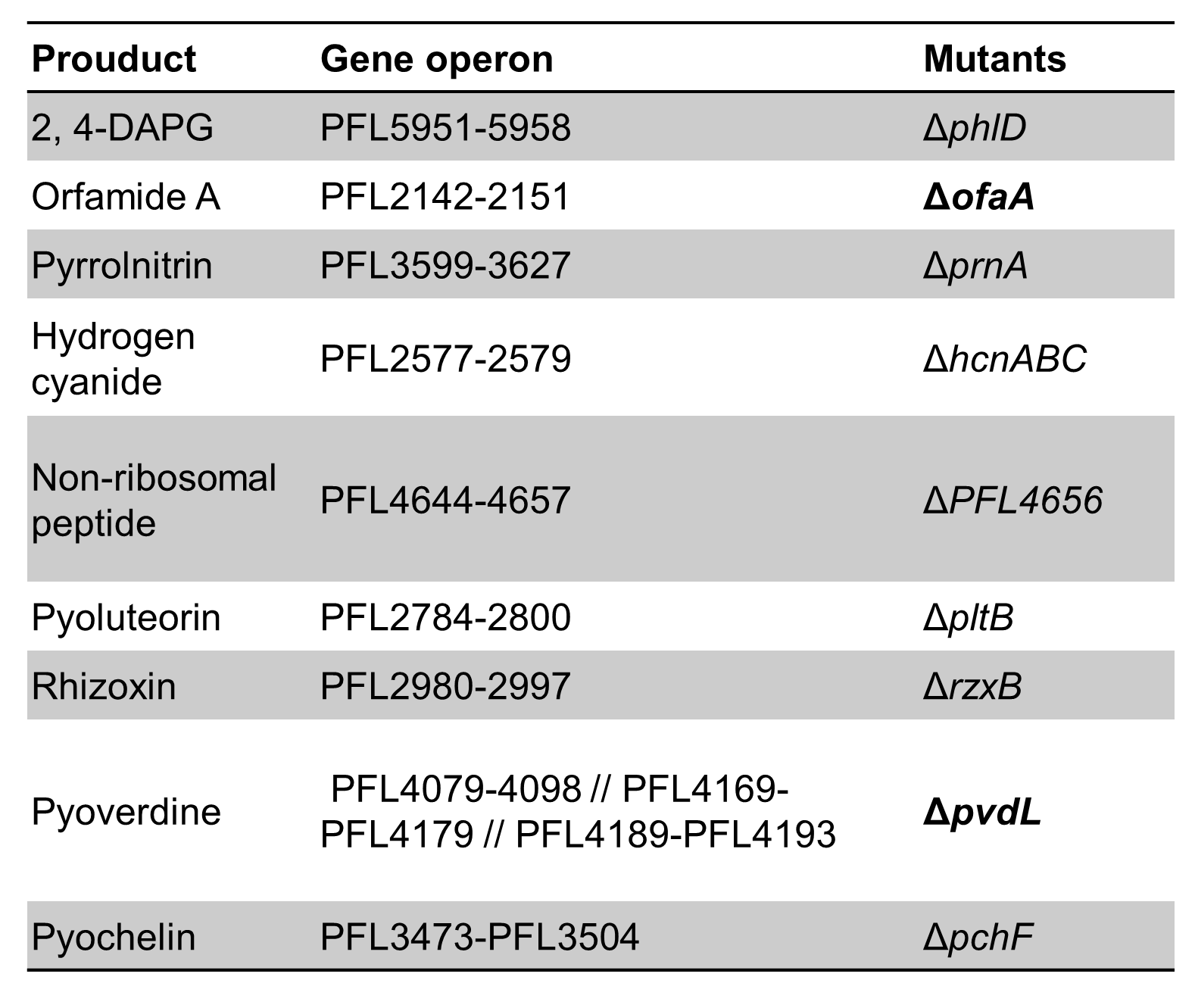


**Figure S1.** Details of selected genes involved in the synthesis of nine antimicrobial metabolites in *P. protegens* Pf-5.


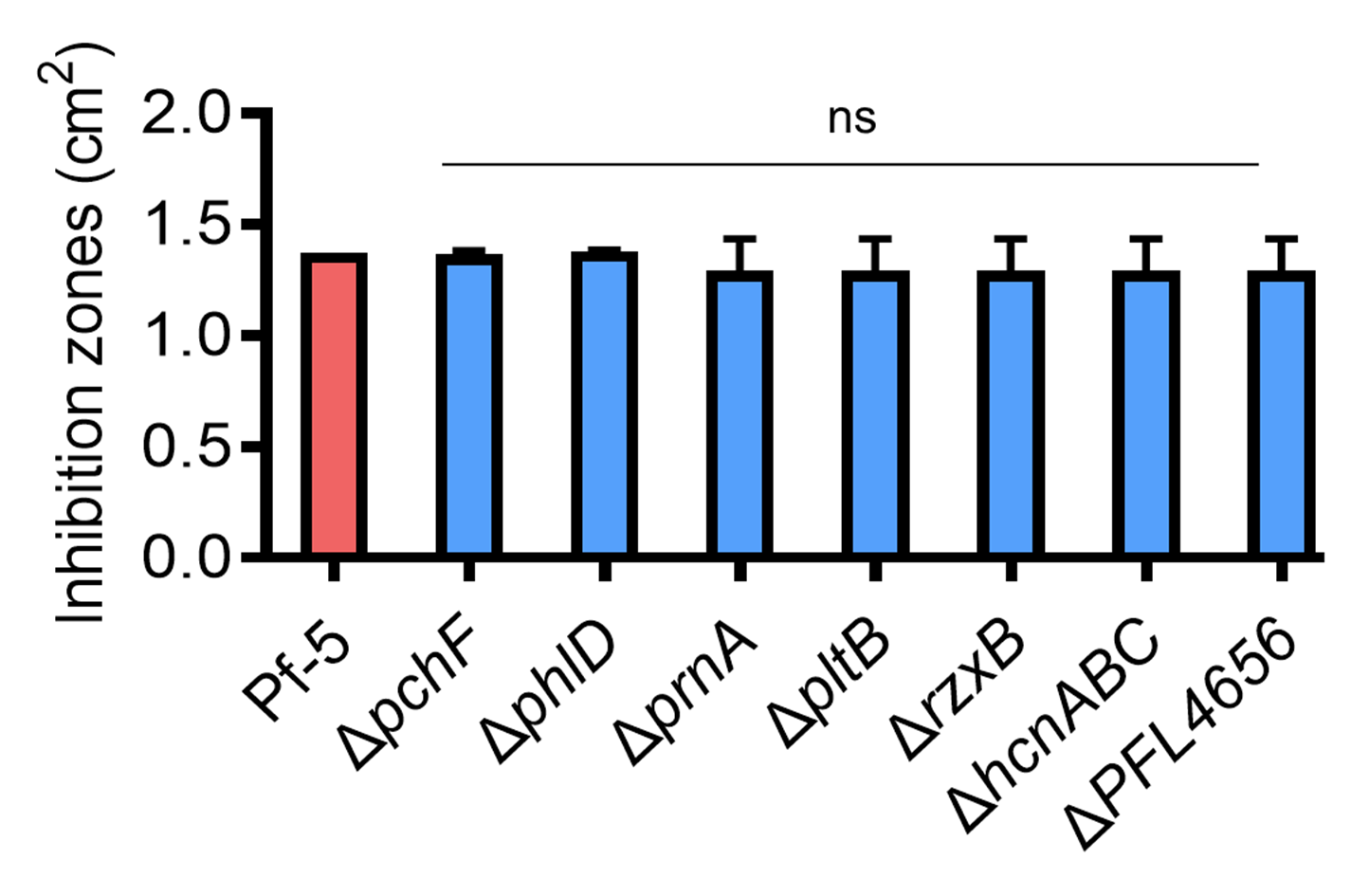


**Figure S2. Antibacterial tests of *P. protegens* mutants listed in Fig. S1 against *L. enzymogenes* OH11 on KB medium.** The results were presented as the mean ± standard deviation (SD) from three independent biological replicates (*n* = 3). Statistical analysis was performed using one-way ANOVA with Tukey's multiple-comparison test. ns, not significant.


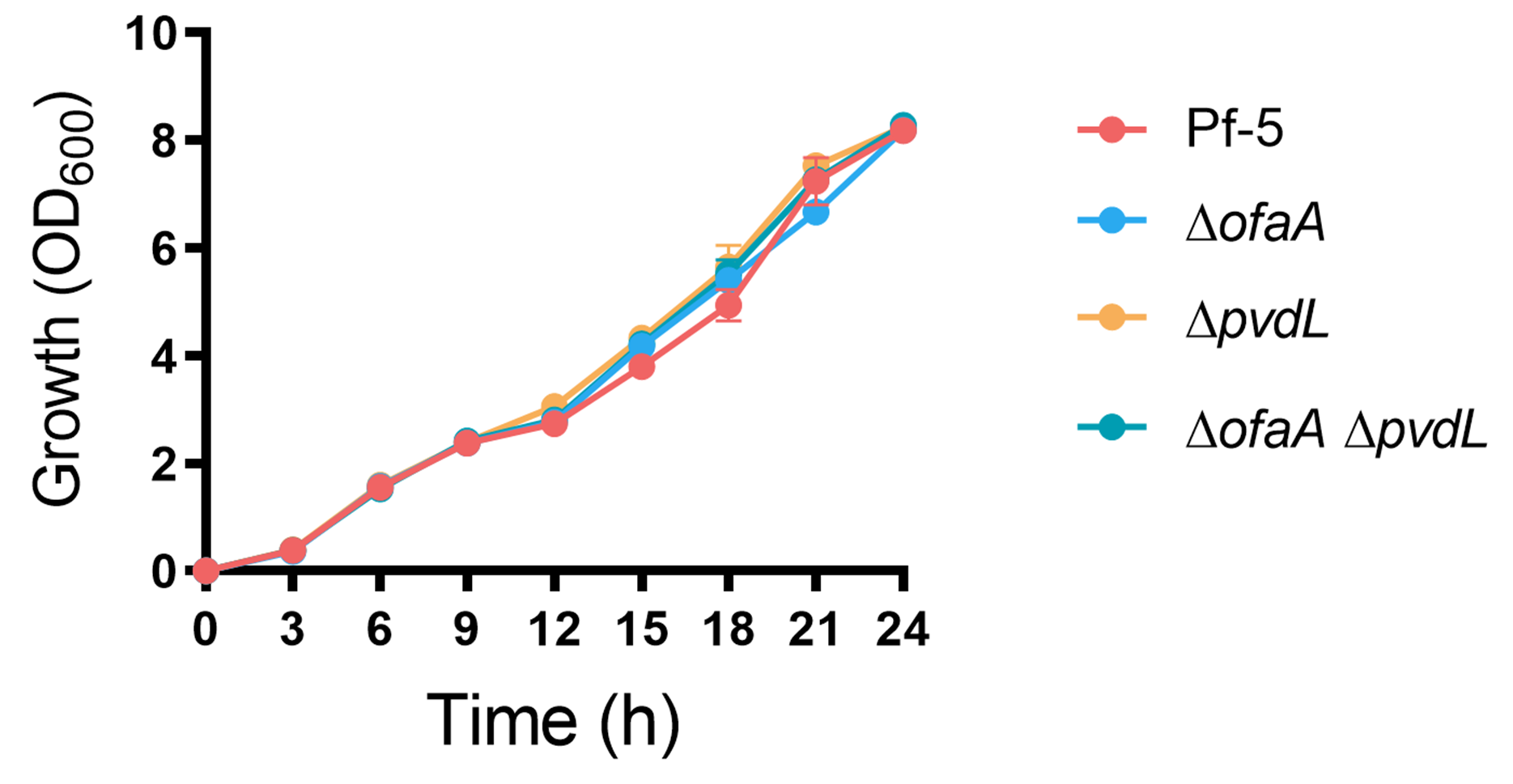


**Figure S3. Growth curves of *P. protegens* Pf-5 and its three derivative mutants in liquid KB medium.** Δ*ofaA*, a mutant strain with an in-frame deletion of *ofaA* that is the first gene of orfamide A biosynthetic operon. Δ*pvdL*, a mutant strain with an in-frame deletion of *pvdL* that is a key member of proverdine biosynthetic operons. Δ*ofaA* Δ*pvdL*, a double mutant of *ofaA* and *pvdL*.


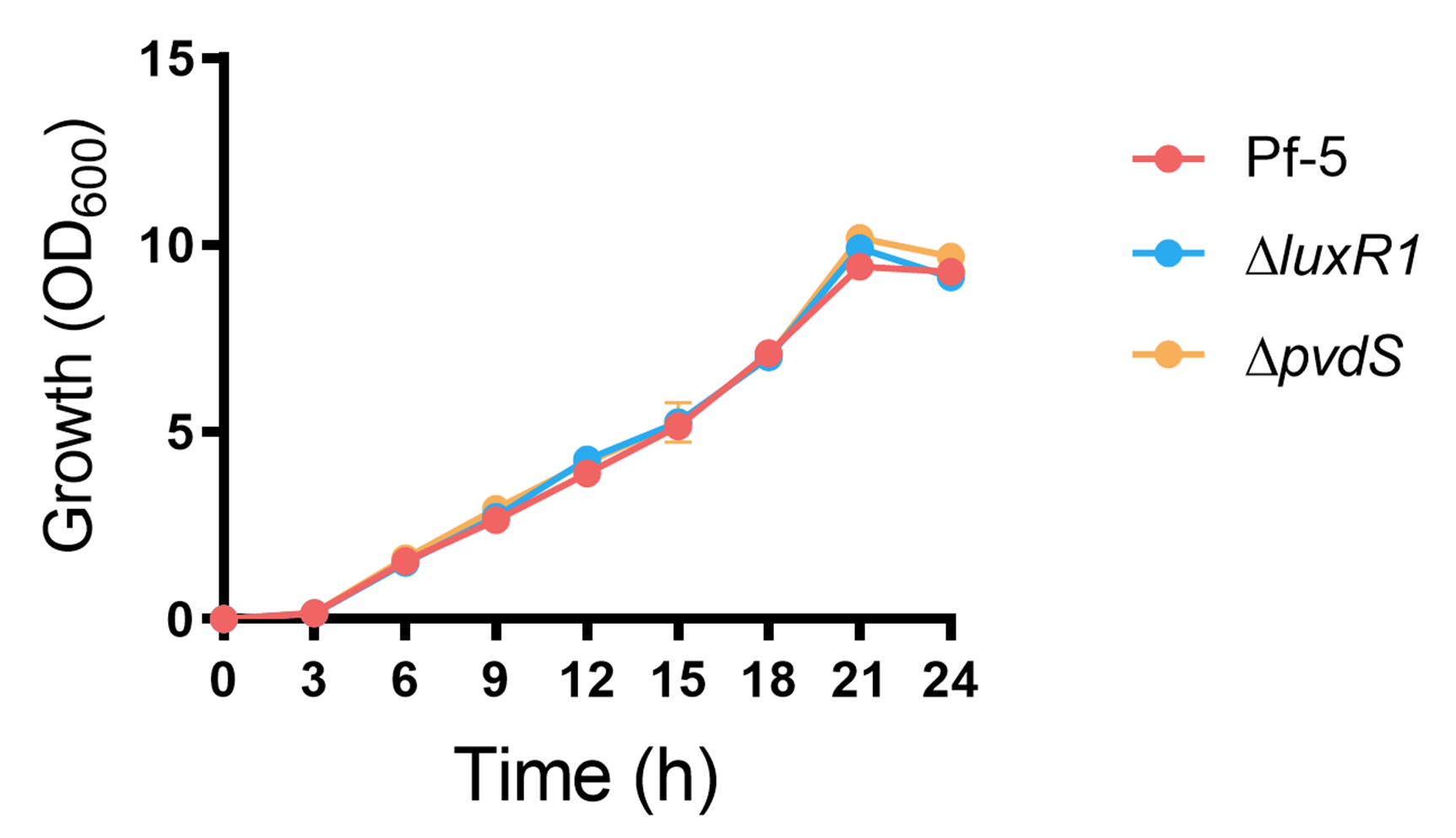


**Figure S4. Growth curves of *P. protegens* Pf-5 and its two derivative mutants in liquid KB medium.** Δ*luxR1*, a mutant strain with an in-frame deletion of *luxR1*. Δ*pvdS*, a mutant strain with a deletion of *pvdS*.


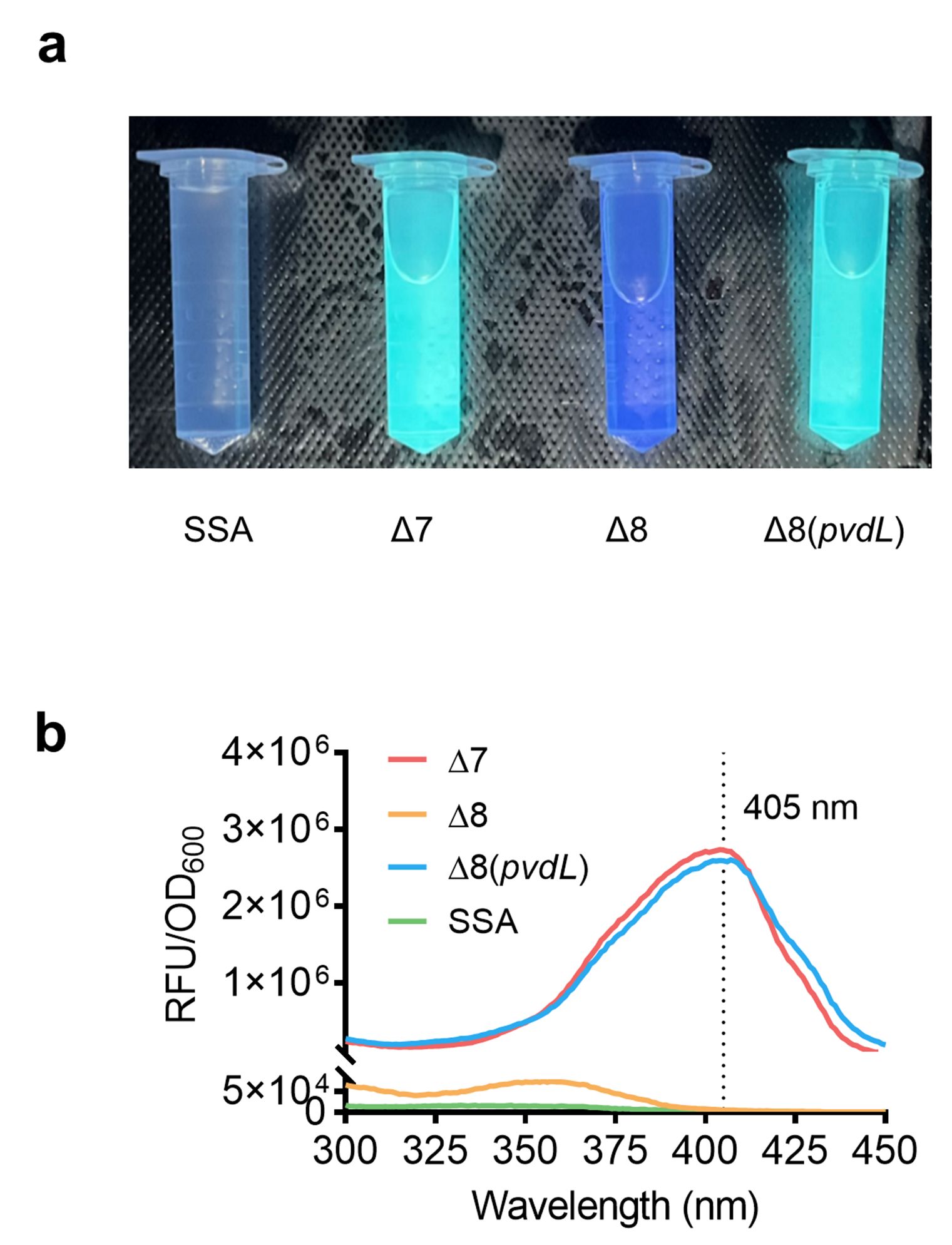


**Figure S5. Fluorescence-based detection of pyoverdine produced by three *P. protegens*-derivative mutants.** (**a**) UV observation of fluorescence intensity in cell-free supernatant produced by *P. protegens*, where green light indicates pyoverdine production. (**b**) Laser scanning quantitative detection of fluorescence intensity in cell-free supernatant produced by *P. protegens*, with excitation wavelength ranging from 300-450nm in 1nm steps and emission wavelength at 480nm. SSA, an iron-deficient culture medium. Δ7, a strain with in-frame deletion of *pltB*, *ofaA*, *prnA*, *hcnABC*, *PFL4656*, *rzxB*, and *phlD* in strain Pf-5. Δ8, a strain with in-frame deletion of *pvdL* in Δ7. Δ8(*pvdL*), a strain with chromosomal complementation of *pvdL* in Δ8 via gene knock-in.


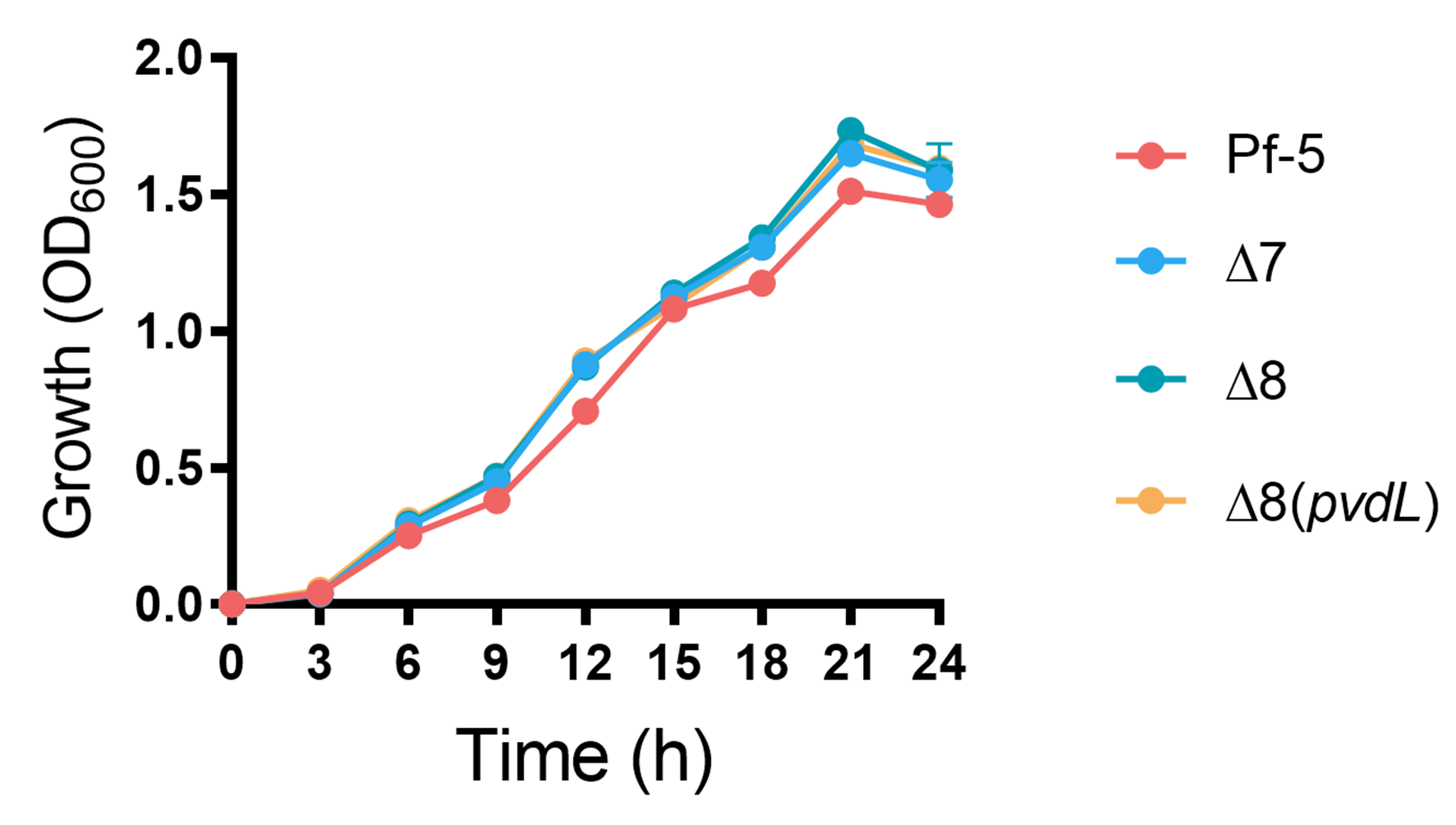


**Figure S6. Growth curves of *P. protegens* Pf-5 and its mutants in** **iron-depleted liquid promoting pyoverdine production.** Δ7, a strain with in-frame deletion of *pltB*, *ofaA*, *prnA*, *hcnABC*, *PFL4656*, *rzxB*, and *phlD* in strain Pf-5. Δ8, a strain with in-frame deletion of *pvdL* inΔ7. Δ8(*pvdL*), a strain with chromosomal complementation of *pvdL* in Δ8 via gene knock-in.


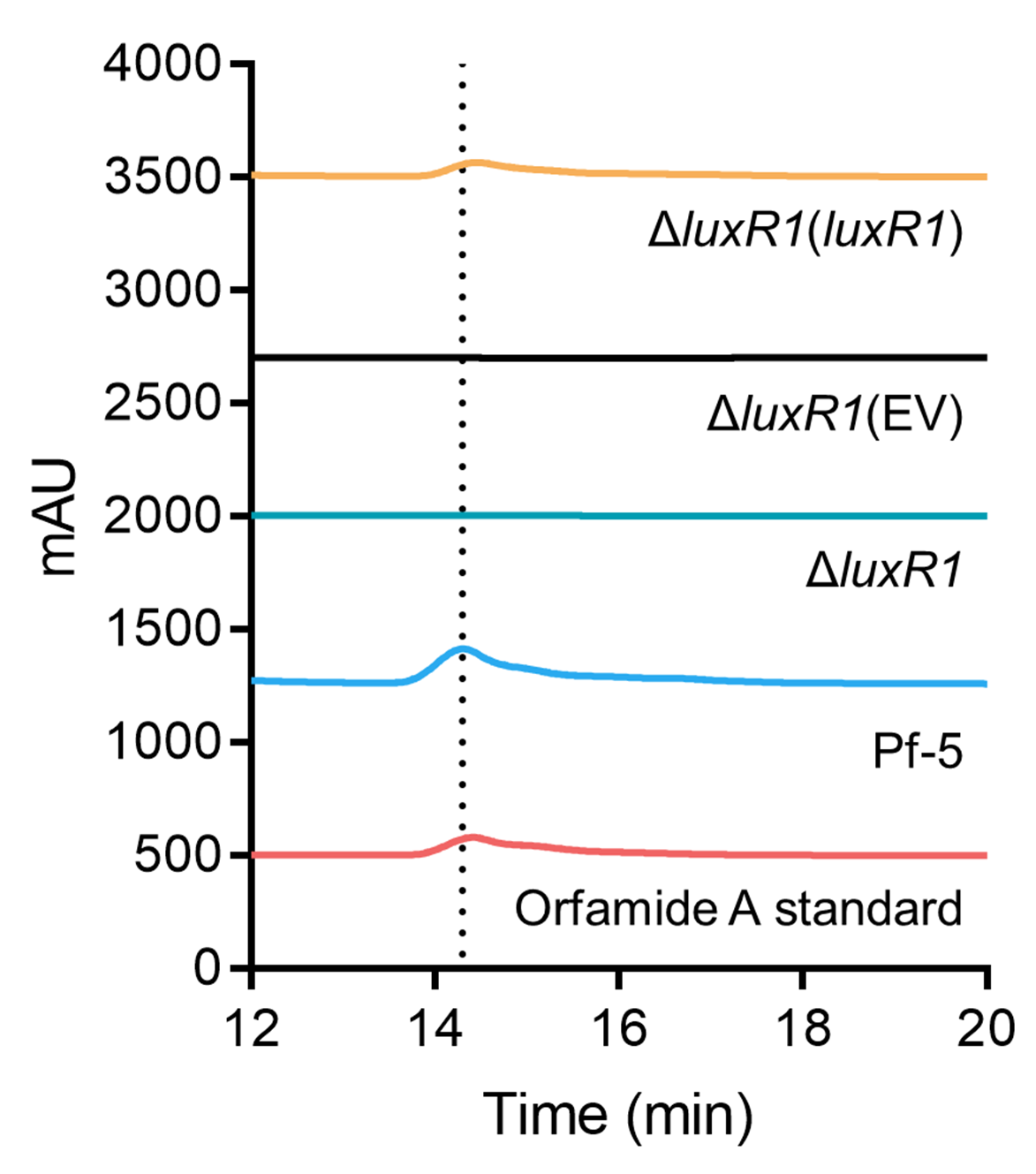


**Figure S7. HPLC-based detection of orfamide A levels in the *luxR1* mutant of *P. protegens* Pf-5.** The red line represents orfamide A standard, and the dashed line indicates the peak time of the orfamide standard. Δ*luxR1*, an in-frame deletion of *luxR1*. Δ*luxR1*(*luxR1*), Δ*luxR1* carrying plasmid-borne *luxR1*. EV stands for empty vector.


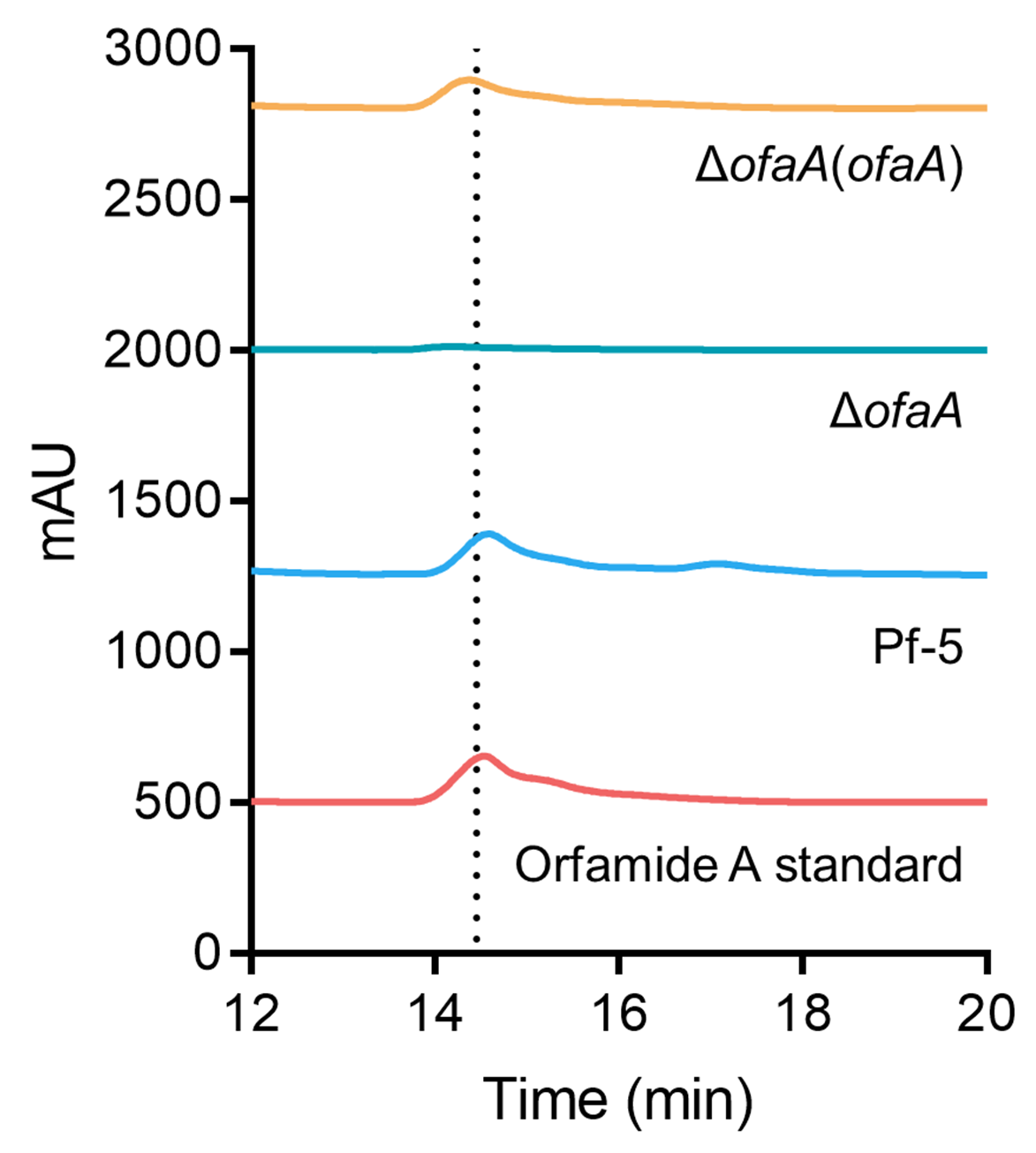


**Figure S8. HPLC-based detection of orfamide A levels in the *ofaA* mutant of *P. protegens* Pf-5.** The red line represents orfamide A standard, and the dashed line indicates the peak time of the orfamide standard. Δ*ofaA*(*ofaA*), a strain with chromosomal complementation of *ofaA* in Δ*ofaA*.


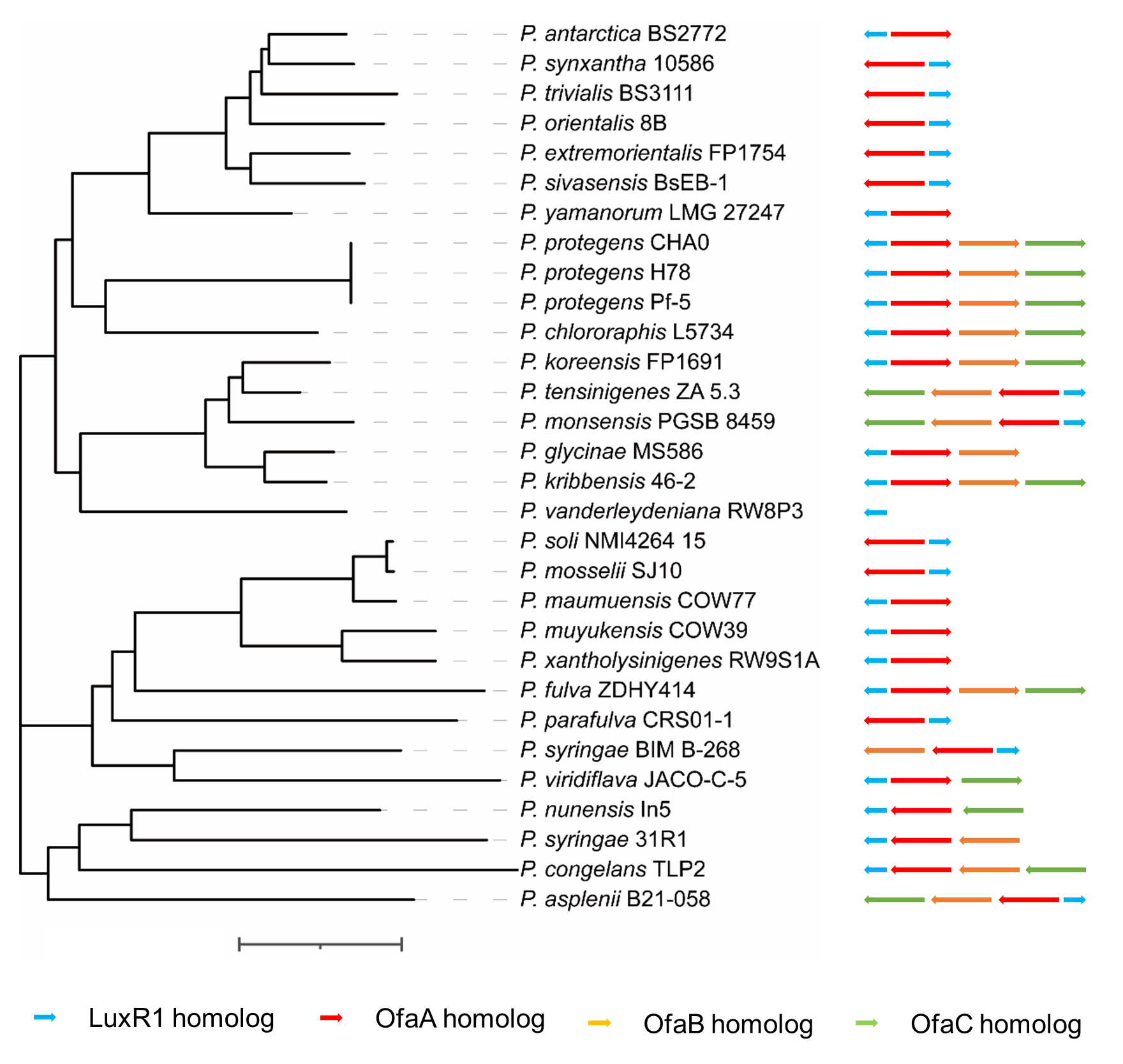


**Figure S9. Phylogenetic analysis of LuxR1 homologs.** A BLAST analysis was conducted using LuxR1 as the query, retrieving 29 homologs. The sequences were aligned, and a Neighbor-Joining phylogenetic tree was constructed with 1,000 bootstrap replicates. The corresponding species and operon structures are depicted on the right, with conserved orfamide A operon genes in color-coded. Tree scale: 0.1.


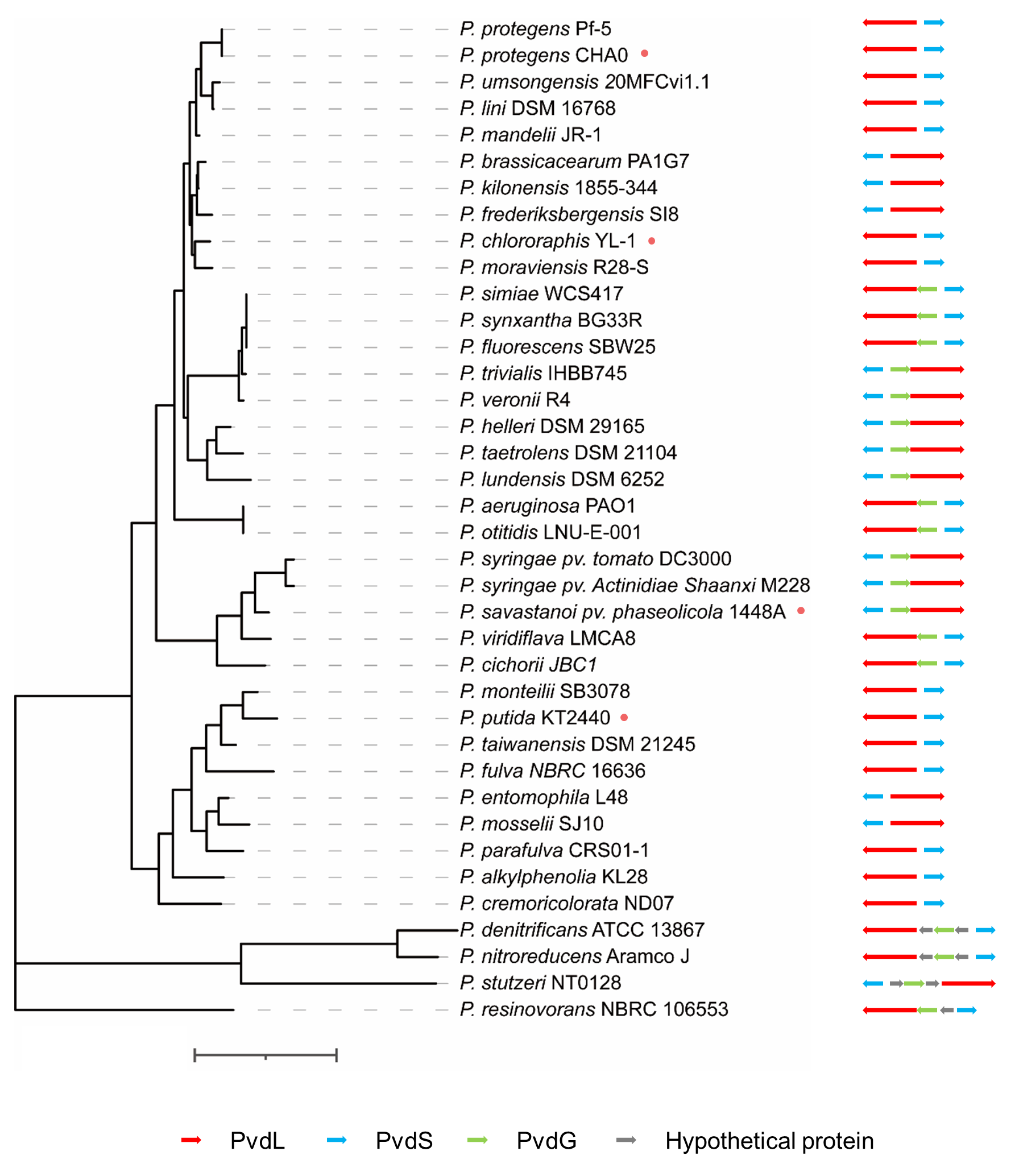


**Figure S10. Phylogenetic analysis of PvdS homologs.** A BLAST analysis was conducted using PvdS as the query, retrieving 37 representative homologs. The sequences were aligned, and a Neighbor-Joining phylogenetic tree was constructed with 1,000 bootstrap replicates. The corresponding species and operon structures are shown on the right, with conserved pyoverdine synthesis proteins in each operon color-coded. Tree scale: 0.1.


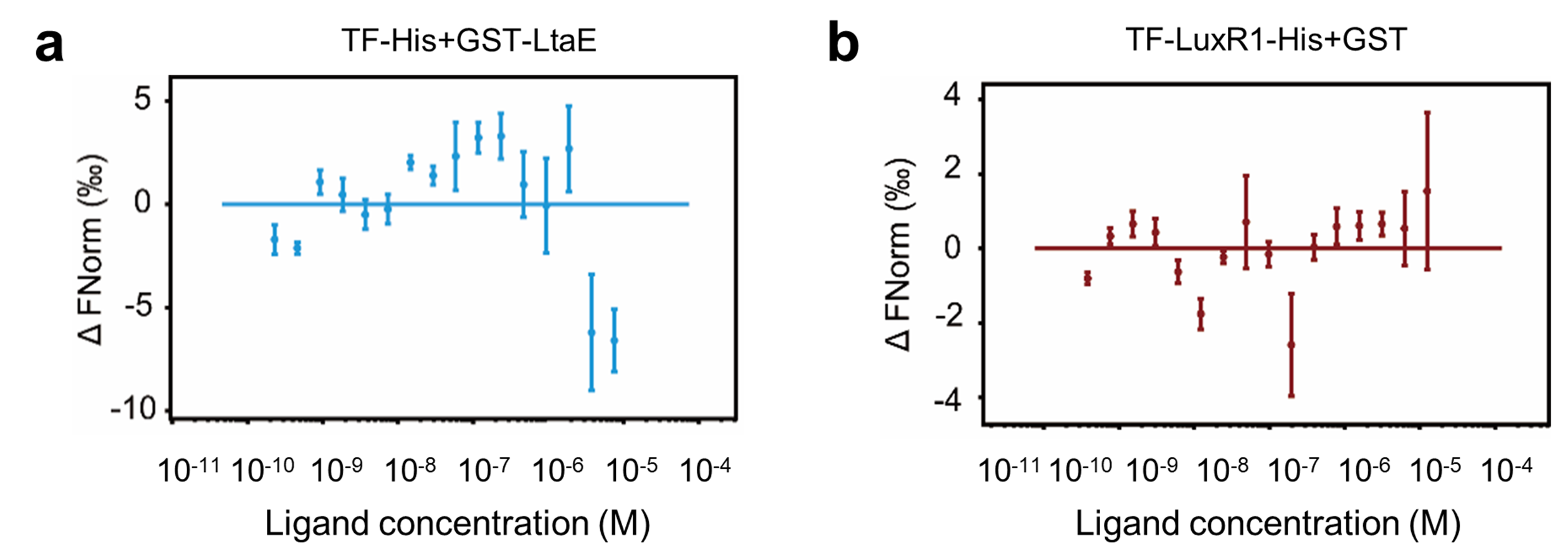


**Figure S11. Microscale thermophoresis showing no interactions between TF-His and GST-LtaE (a), and between TF-LuxR1-His and GST (b).**


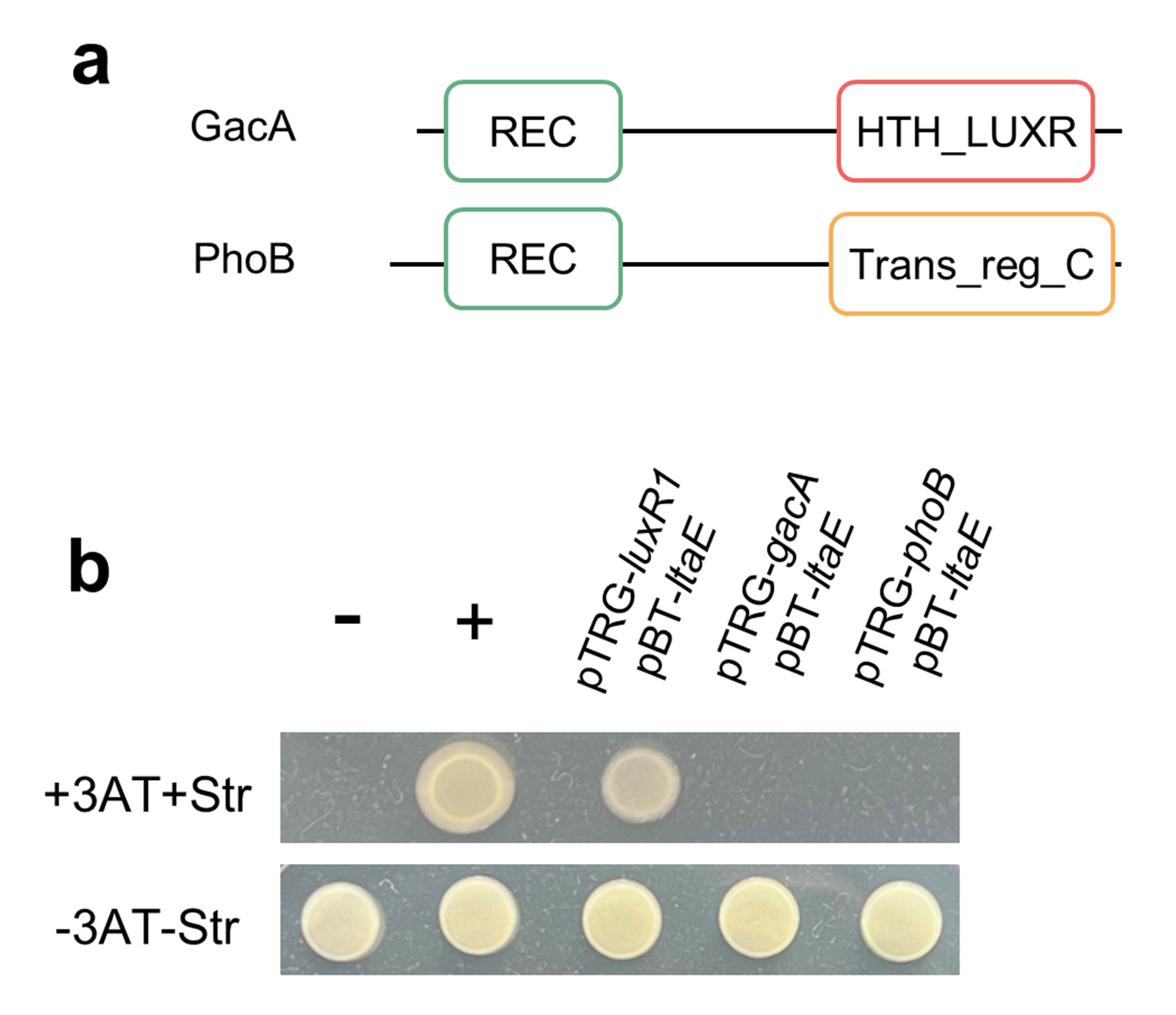


**Figure S12. B2H shows no interactions between LtaE and two transcriptional factors from *P. protegens* Pf-5. (a)** Schematic model of two additional transcription factors. HTH_LUXR refers to the helix-turn-helix (HTH) DNA-binding domain found in the LuxR-type transcriptional regulators; REC, refers to the Response Regulator Receiver domain; Trans_reg_C refers to the Transcriptional Regulatory Domain (Trans_reg) with a C-terminal. GacA Locus Tag: PFL3563; PhoB Locus Tag: PFL6108. (**b**) B2H shows that LtaE interacted with LuxR1 but not with the two transcription factors listed in **Fig. S12a**. Successful interaction between the two proteins was determined by the growth of transformed *E.coli* strain carrying both vectors on histidine-deficient medium supplemented with 5mM 3-AT and 2 μg/mL Str. "-" represents the negative control; "+" represents the positive control; "+3AT+Str" indicates the addition of 3-AT and Str; "-3AT-Str" indicates the absence of 3-AT and Str.


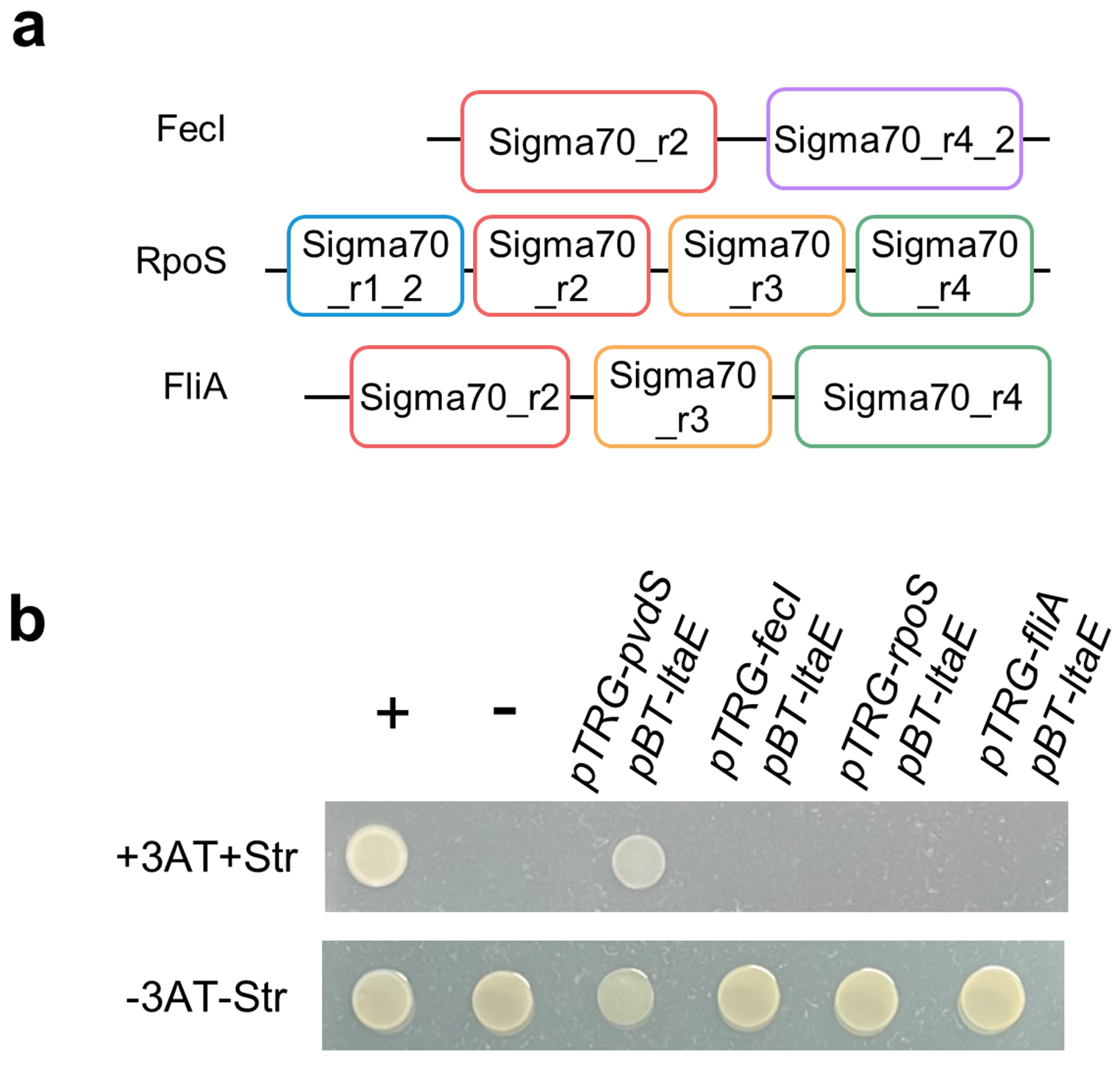


**Figure S13. B2H shows no interactions between LtaE and three sigma factors from *P. protegens* Pf-5.** (**a**) Schematic model of three additional sigma factors. Sigma70_r2 refers to the Sigma-70 factor, region 2; Sigma70_42 refers to the Sigma-70 factor, region 4; Sigma70_r4_2 refers to the Sigma-70 factor, region 4.2; Sigma70_r1_2 refers to the Sigma-70 factor, region 1.2; Sigma70_r3 refers to the Sigma-70 factor, region 3. FecI Locus Tag: PFL0984; RpoS Locus Tag: PFL1207; FliA Locus Tag: PFL1667. (**b**) B2H shows that LtaE did not interact with the three sigma factors listed in **Fig. S13a**. Successful interaction between the two proteins was determined by the growth of transformed *E.coli* strain carrying both vectors on histidine-deficient medium supplemented with 5mM 3-AT and 2 μg/mL Str. "-" represents the negative control; "+" represents the positive control; "+3AT+Str" indicates the addition of 3-AT and Str; "-3AT-Str" indicates the absence of 3-AT and Str.


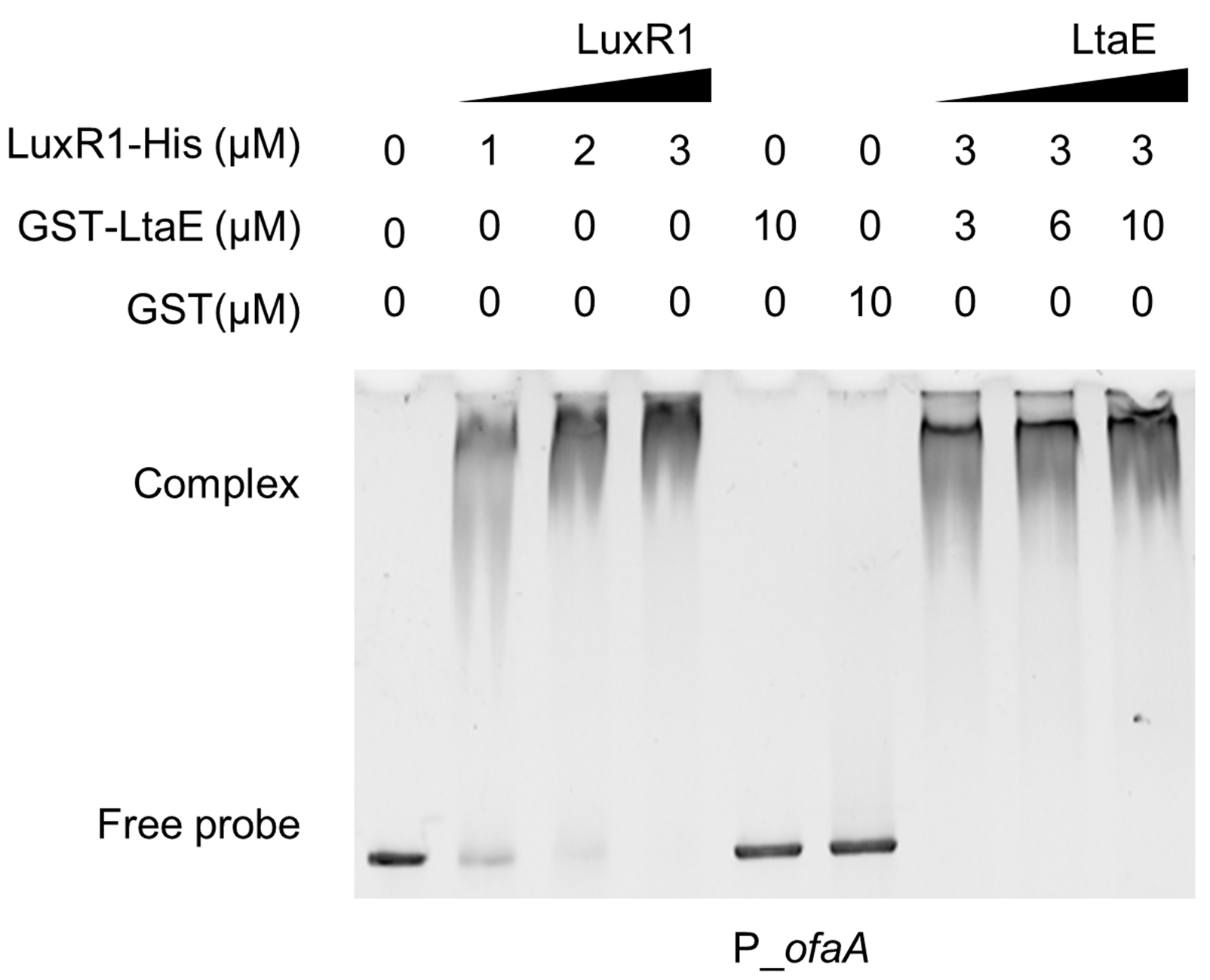


**Figure S14. EMSA shows that LtaE did not affect the binding strength of LuxR1 to the *ofaA* gene promoter.**

**
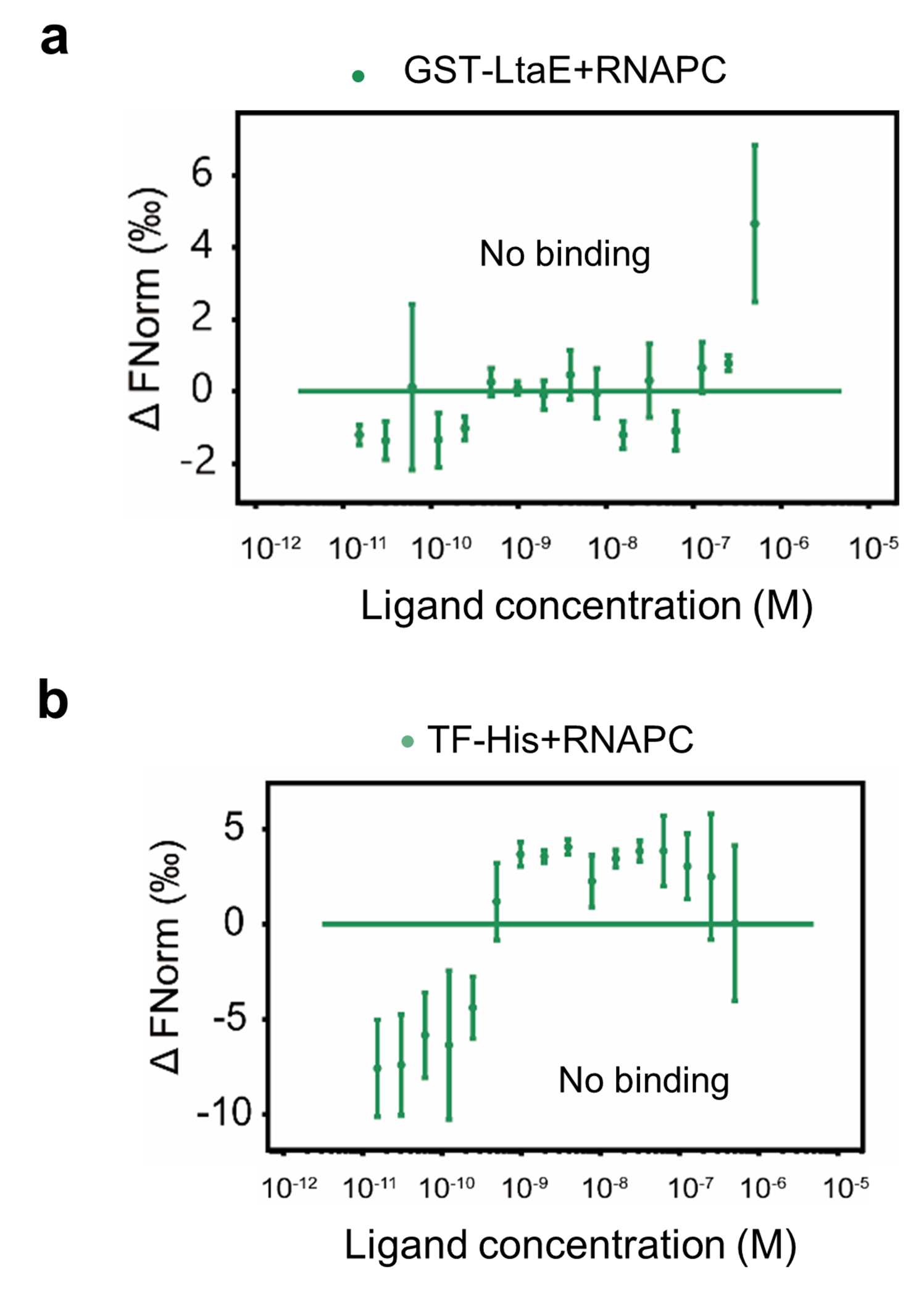
**

**Figure S15. MST shows no interactions between RNA polymerase core enzyme (RNAPC) with GST-LtaE (a) and TF-His (b)**. TF stands for Trigger Factor, a ribosome-associated chaperone from *E. coli*. RNAPC stands for the commercial RNA polymerase core enzyme.


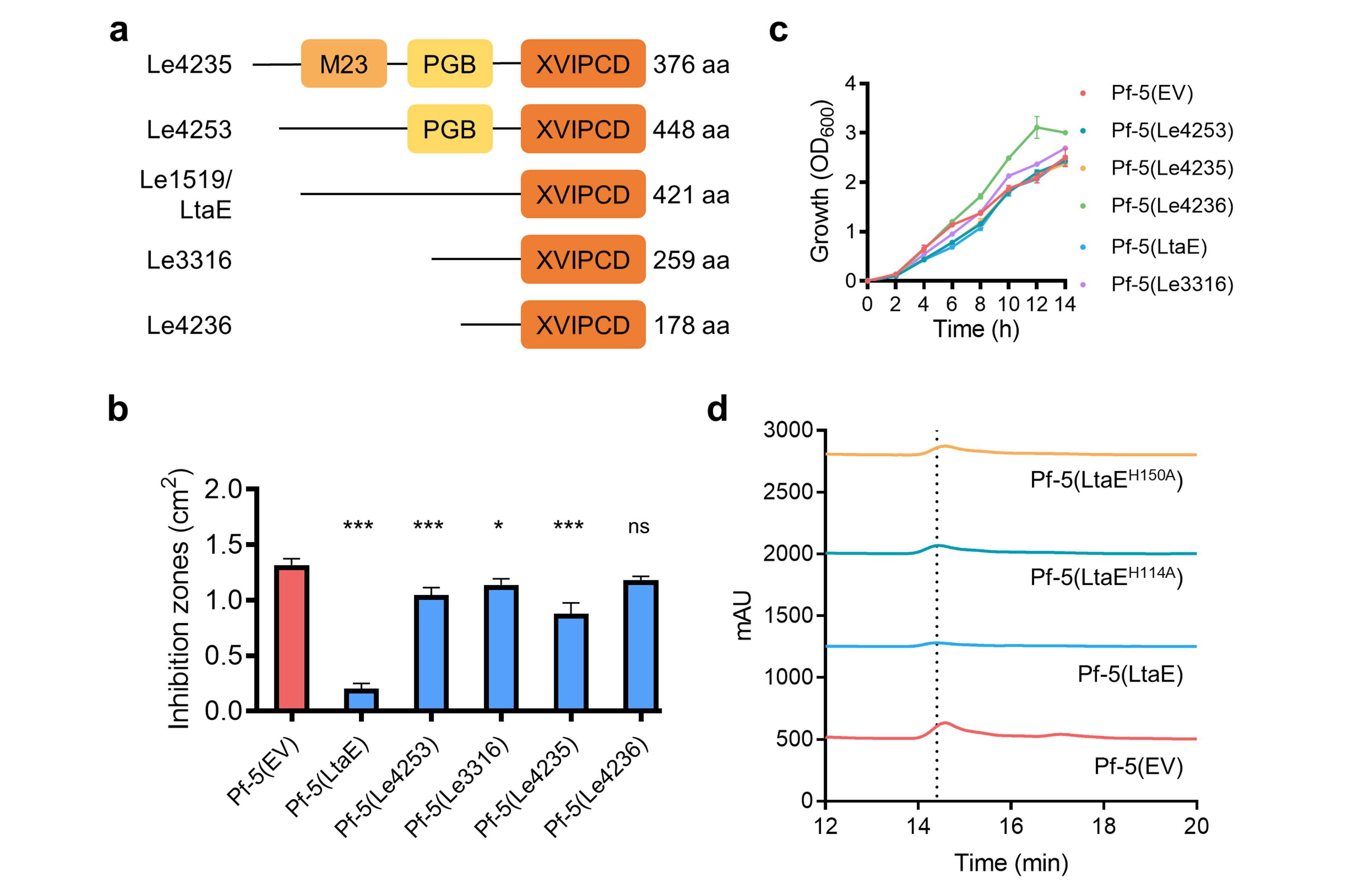


**Figure S16. Expression of *ltaE* inhibits orfamide A production in *P. protegens* Pf-5.** (**a**) Schematic model of five predicted T4SS effectors (T4Es) in *L. enzymogenes* OH11. M23, peptidase family M23. PGB, peptidoglycan binding domain. XVIPCD, *Xanthomonas* VirD4-interacting protein conserved domain. (**b**) Antibacterial test of *P. protegens* Pf-5 expressing each of the five T4E genes listed in Fig. S15a against strain OH11. EV stands for empty vector pBBR1MCS-5. One-way ANOVA with Tukey’s multiple-comparison test compared all samples to the Pf-5(EV) strain. ****P* < 0.001; **P* < 0.05; ns, not signiﬁcant. Results are expressed as mean ± SD from three independent biological replicates (*n* = 3). (**c**) Growth curve analysis of *P. protegens* Pf-5 expressing each of the five T4E genes listed in Fig. S15a**.** EV stands for empty vector pBBR1MCS-5. (**d**) Expression of *ltaE* in *P. protegens* Pf-5 reduced orfamide A production determined by HPLC. EV stands for empty vector pBBR1MCS-5. LtaE^H114A^ and LtaE^W150A^ are two reported LtaE variants whose expression in *P. protegens* Pf-5 could not trigger activated antifungal effects under PDA plates^1^.

**
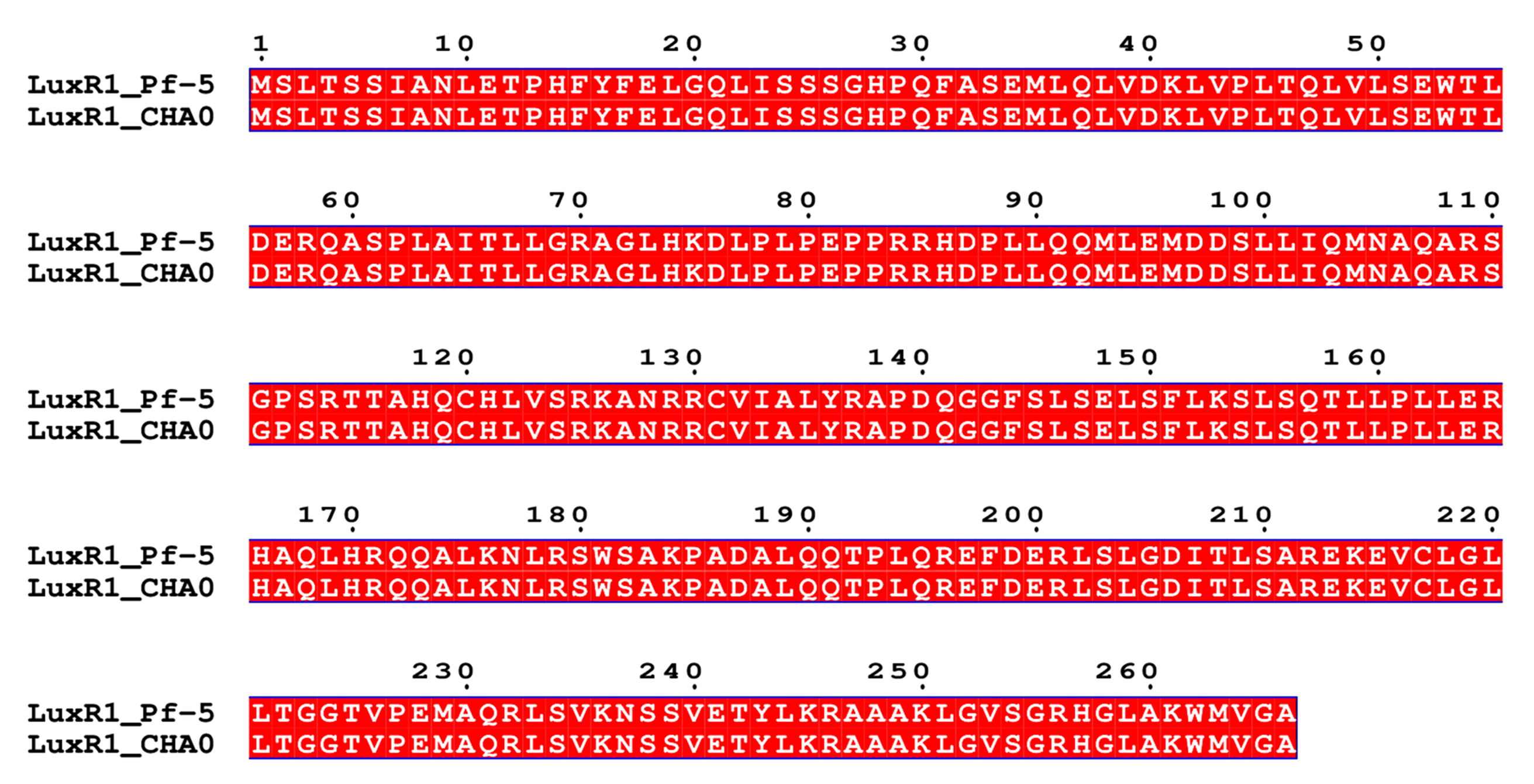
**

**Figure S17. Sequence alignment for LuxR1 from *P. protegens* Pf-5 and CHA0.** The alignment, performed using the Needleman-Wunsch algorithm, indicates a complete identity between the amino acid sequences.

**
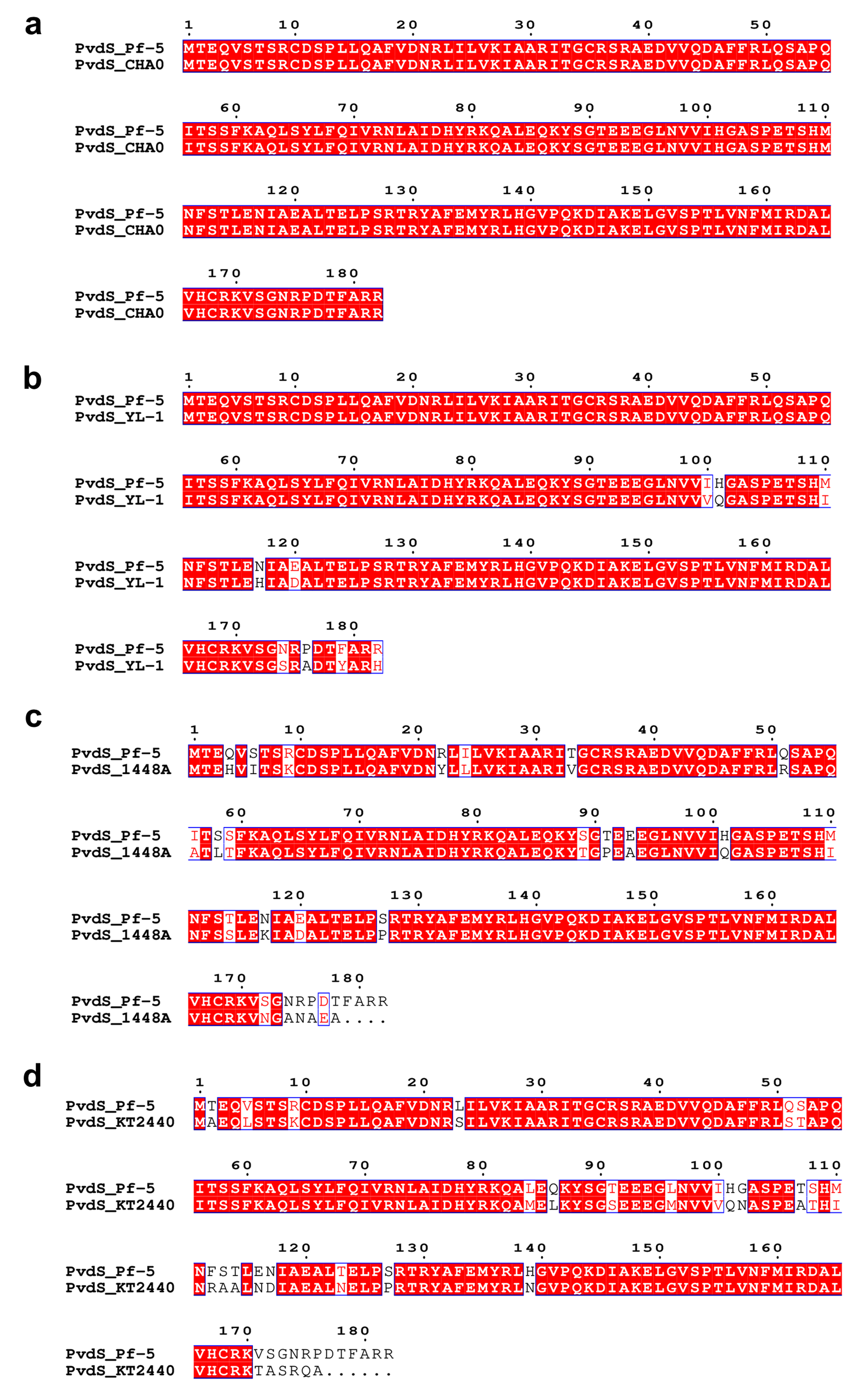
**

**Figure S18. Sequence alignment for PvdS from *P. protegens* Pf-5 and other *Pseudomonas* strains.** Alignments were performed using the Needleman-Wunsch algorithm. CHA0, *Pseudomonas protegens*; YL-1, *P. chlororaphis*; 1448A, *P. syringae*; KT2440, *P. putida*.

**
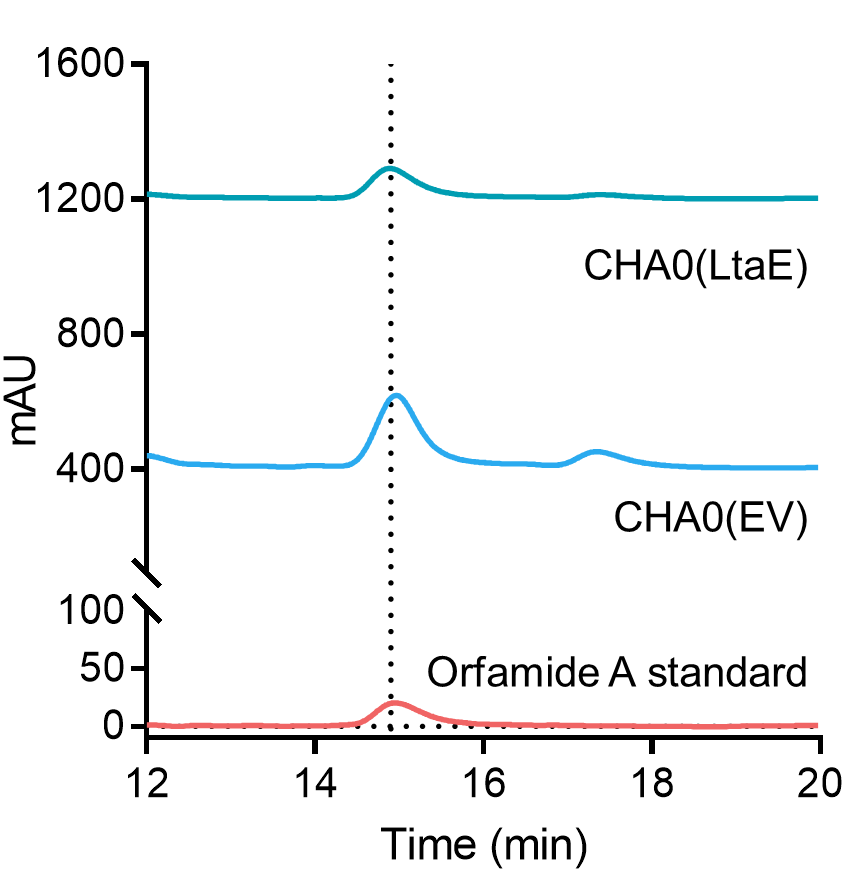
**

**Figure 19. Expression of *ltaE* in *P. protegens* CHA0 reduced orfamide A production determined by HPLC.** The red line represents orfamide A standard, and the dashed line indicates the peak time of the orfamide standard. EV stands for empty vector.

**
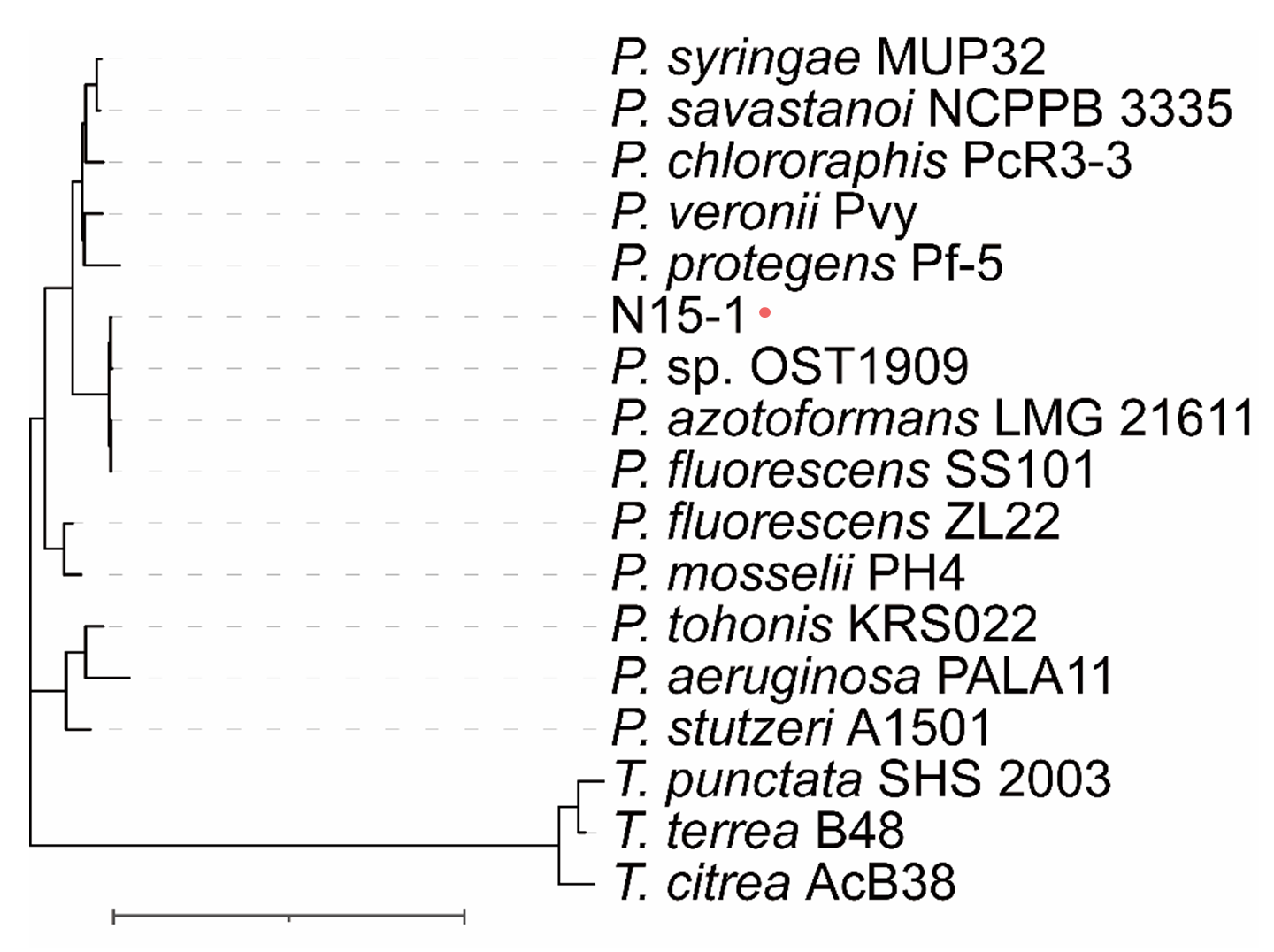
**

**Figure S20. Phylogenetic analysis of strain N15-1 based on *16S rRNA* gene sequences.** A maximum-likelihood tree illustrating the evolutionary relationships between strain N15-1 and its closely related strains. The tree was inferred from *16S rRNA* gene sequences using 1,000 bootstrap replicates. Tree scale: 0.1.


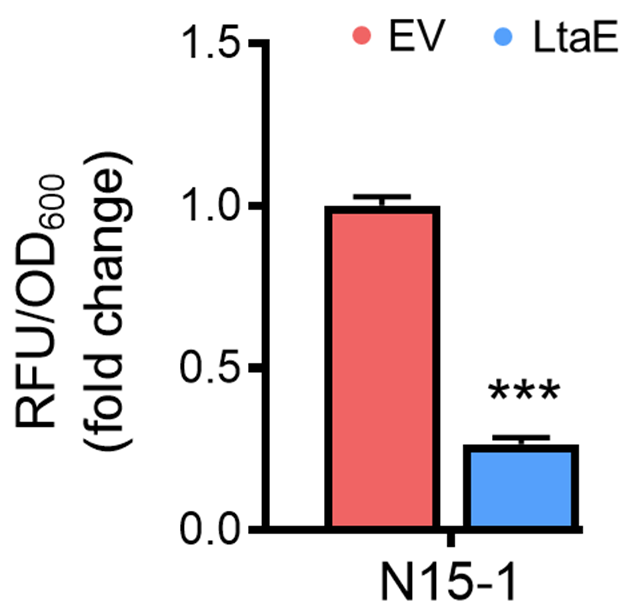


**Figure S21. Quantification of proverdine yield (fold changes) in *Pseudomonas* N15-1 expressing *ltaE*.** ****P* < 0.001, unpaired two-tailed Welch’s *t*-test. Results are expressed as mean ± SD.


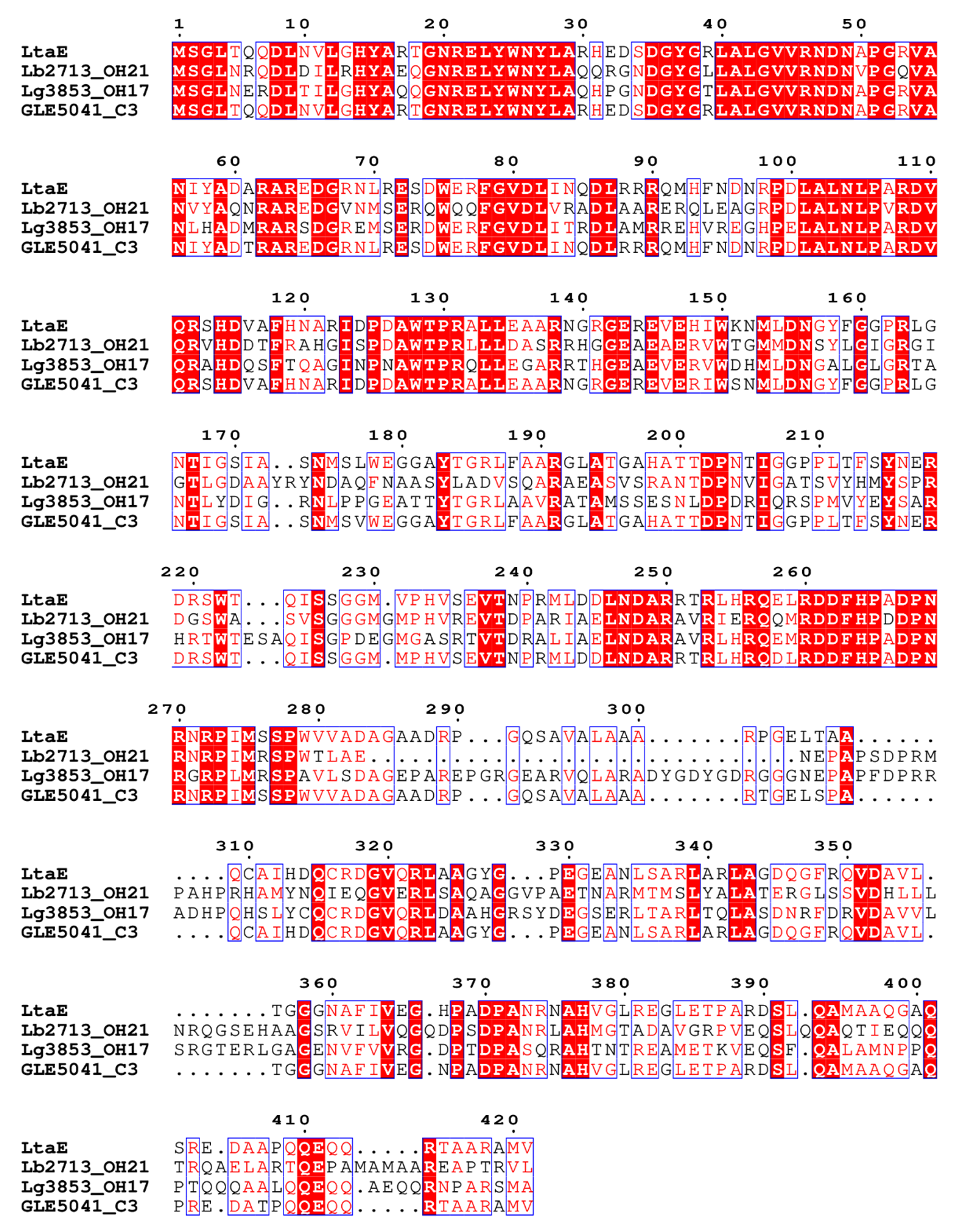


**Figure S22. Multiple sequence alignment of LtaE and its homologs from different *Lysobacter* strains.** The alignment includes LtaE from *L. enzymogenes* OH11, Lb2713 from *L. brunescens* OH21, Lg3853 from *L. gummosus* OH17, and GLE5041 from *L. enzymogenes* C3. The sequences were aligned using the MUSCLE algorithm, and the conservation of residues is visualized with ESPript 3.0. Highly conserved residues are highlighted with red text, while similar residues are denoted in blue boxes.

**
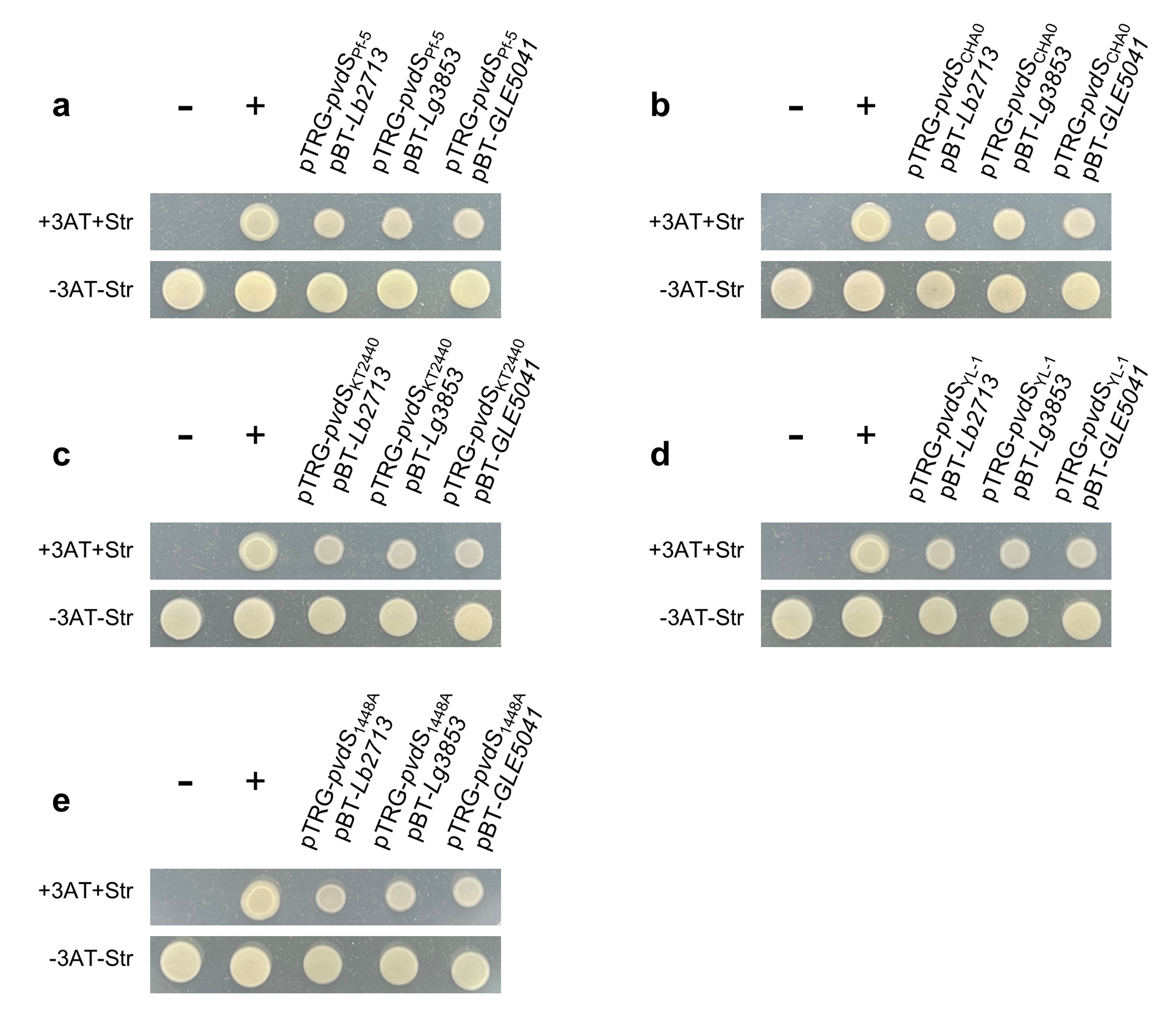
**

**Figure S23. Bacterial two-hybrid assays showing protein-protein interactions between LtaE homologs and PvdS homologs across different *Lysobacter* and *Pseudomonas* strains. (a)** *P. protegens* Pf-5, **(b)** *P. protegens* CHA0, **(c)** *P. putida* KT2440, **(d)** *P. chlororaphis* YL-1, and **(e)** *P. syringae* 1448A. Successful interaction between the two proteins was determined by the growth of transformed *E.coli* strain carrying both vectors on histidine-deficient medium supplemented with 5mM 3-AT and 2 μg/mL Str. "-" represents the negative control; "+" represents the positive control; "+3AT+Str" indicates the addition of 3-AT and Str; "-3AT-Str" indicates the absence of 3-AT and Str.

**
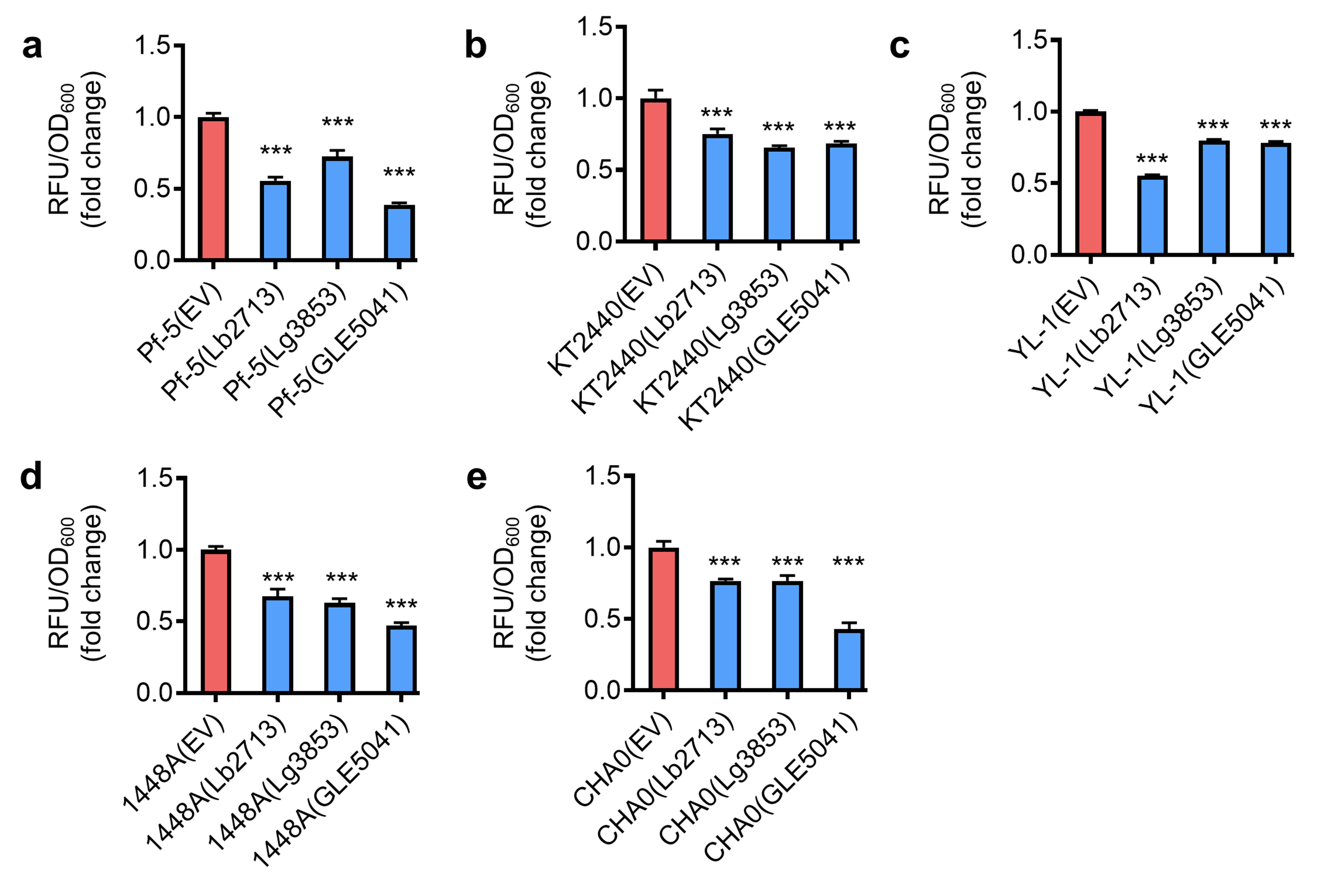
**

**Figure S24. Quantification of proverdine yield (fold changes) in five *Pseudomonas* strains expressing *ltaE* homologs. (a)** *P. protegens* Pf-5, **(b)** *P. putida* KT2440, **(c)** *P. chlororaphis* YL-1, **(d)** *P. syringae* 1448A, and **(e)** *P. protegens* CHA0. One-way ANOVA with Dunnett’s multiple-comparison test was employed. ****P* < 0.001. Results were expressed as mean ± SD from three independent biological replicates (*n* = 3).


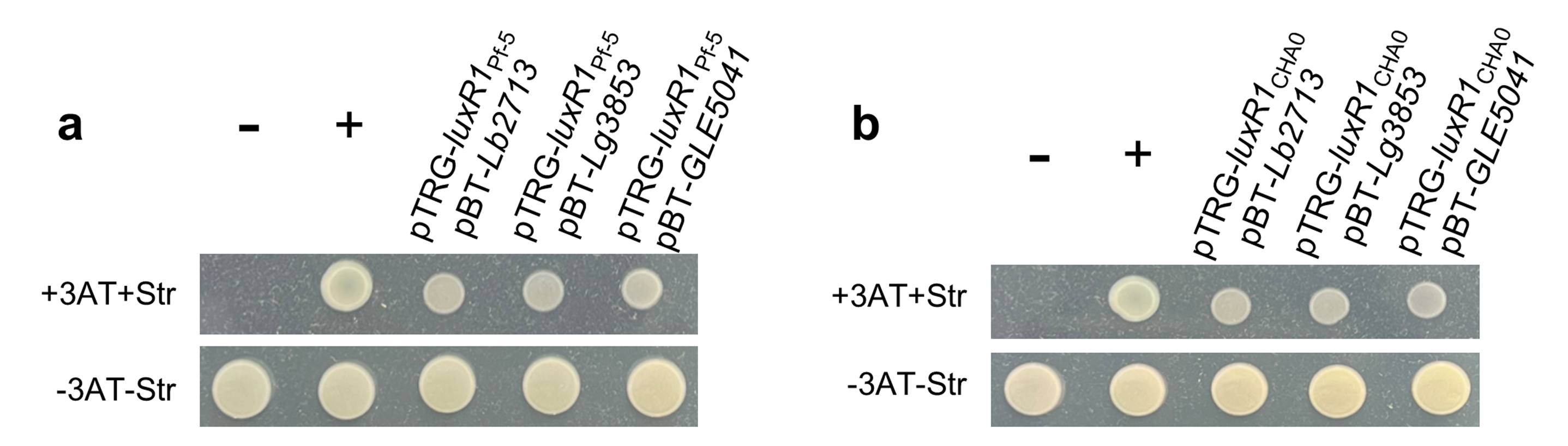


**Figure S25. Bacterial two-hybrid assay interactions.** **(a)** Interactions between LtaE homologs and LuxR1 from *P. protegens* Pf-5. **(b)** Interactions between LtaE homologs and LuxR1 homolog from *P. protegen****s*** CHA0. Successful interaction between the two proteins was determined by the growth of transformed *E.coli* strain carrying both vectors on histidine-deficient medium supplemented with 5mM 3-AT and 2 μg/mL Str. "-" represents the negative control; "+" represents the positive control; "+3AT+Str" indicates the addition of 3-AT and Str; "-3AT-Str" indicates the absence of 3-AT and Str.

**
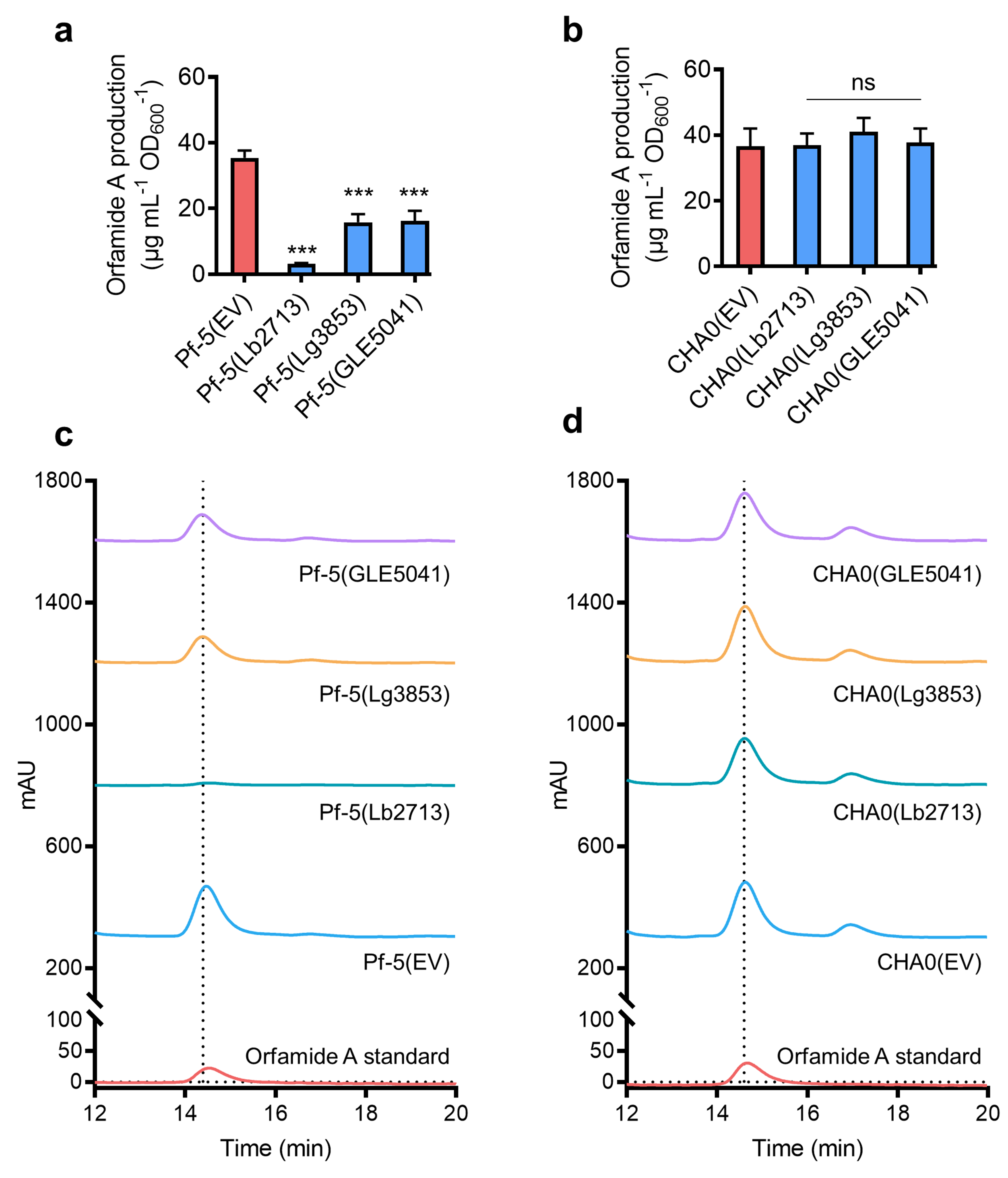
**

**Figure S26. Effect of LtaE homologs on orfamide A production in *P. protegens* pf-5 and CHA0. (a-b)** Inhibition of orfamide A production by LtaE homologs was observed in strain Pf-5 **(a)** but not in strain CHA0 **(b)**. One-way ANOVA with Dunnett’s multiple-comparison test was employed. ****P* < 0.001; ns, not signiﬁcant. Results were expressed as mean ± SD from three independent biological replicates (*n* = 3). **(c-d)** HPLC-based detection of orfamide A levels in strains Pf-5 **(c)** and CHA0 **(d)** expressing *ltaE* homologous genes. The red line represents orfamide A standard, and the dashed line indicates the peak time of the orfamide standard. EV stands for an empty vector.

**Reference**

1. Wang B, Zhang Z, Xu F, Yang Z, Li Z, Shen D, et al. Soil bacterium manipulates antifungal weapons by sensing intracellular type IVA secretion system effectors of a competitor. ISME J. 2023;17:2232-46.
